# Difference in Gastrointestinal Cancer Risk and Mortality by Dietary Pattern Analysis: A Systematic Review and Meta-Analysis

**DOI:** 10.1093/nutrit/nuae090

**Published:** 2024-07-17

**Authors:** Zegeye Abebe, Molla Mesele Wassie, Tefera Chane Mekonnen, Amy C Reynolds, Yohannes Adama Melaku

**Affiliations:** Flinders Health and Medical Research Institute, Flinders University, Adelaide, South Australia, Australia; Department of Human Nutrition, Institute of Public Health, College of Medicine and Health Sciences, University of Gondar, P. O. Box 196, Gondar, Ethiopia; Flinders Health and Medical Research Institute, Flinders University, Adelaide, South Australia, Australia; Adelaide Medical School, The University of Adelaide, South Australian Health and Medical Research Institute, North Terrace, South Australia, Australia; Flinders Health and Medical Research Institute, Flinders University, Adelaide, South Australia, Australia; Flinders Health and Medical Research Institute, Flinders University, Adelaide, South Australia, Australia; Cancer Epidemiology Division, Cancer Council Victoria, Melbourne, Australia

**Keywords:** colorectal cancer, dietary patterns, principal component analysis, reduced rank regression, gastrointestinal, neoplasm

## Abstract

**Context:**

Several studies have demonstrated that dietary patterns identified by a posteriori and hybrid methods are associated with gastrointestinal (GI) cancer risk and mortality. These studies applied different methods for analyzing dietary data and reported inconsistent findings.

**Objective:**

This systematic review and meta-analysis were aimed to determine the association between dietary patterns, derived using principal component analysis (PCA) and reduced rank regression (RRR), and GI cancer risk and GI cancer–caused mortality.

**Data Source:**

Articles published up to June 2023 in English were eligible for inclusion. The Medline, SCOPUS, Cochrane Library, CINHAL, PsycINFO, ProQuest, and Web of Sciences databases were used to identify prospective studies. The Preferred Reporting Item for Systematic Review and Meta-analysis Protocol 2020 was used to report results.

**Data Extraction:**

A total of 28 studies were eligible for inclusion. Varied approaches to deriving dietary patterns were used, including PCA (n = 22), RRR (n = 2), combined PCA and RRR (n = 1), cluster analysis (CA; n = 2) and combined PCA and CA (n = 1).

**Data Analysis:**

Two dietary patterns, “healthy” and “unhealthy,” were derived using PCA and RRR. The healthy dietary pattern was characterized by a higher intake of fruits, whole grains, legumes, vegetables, milk, and other dairy products, whereas the unhealthy dietary pattern was characterized by a higher intake of red and processed meat, alcohol, and both refined and sugar-sweetened beverages. The findings indicated that the PCA-derived healthy dietary pattern was associated with an 8% reduced risk (relative risk [RR], 0.92; 95% CI, 0.87-0.98), and the unhealthy dietary pattern was associated with a 14% increased risk (RR, 1.14; 95% CI, 1.07-1.22) of GI cancers. Similarly, the RRR-derived healthy dietary pattern (RR, 0.83; 95% CI, 0.61-1.12) may be associated with reduced risk of GI cancers. In contrast, the RRR-derived unhealthy dietary pattern (RR, 0.93; 95% CI, 0.57-1.52) had no association with a reduced risk of GI cancers. Similarly, evidence suggested that PCA-derived healthy dietary patterns may reduce the risk of death from GI cancers, whereas PCA-derived unhealthy dietary patterns may increase the risk.

**Conclusion:**

Findings from prospective studies on the association of PCA-derived dietary patterns and the risk of GI cancers support the evidence of healthy and unhealthy dietary patterns as either protective or risk-increasing factors for GI cancers and for survivorship, respectively. The findings also suggest that the RRR-derived healthy dietary pattern reduces the risk of GI cancers (albeit with low precision), but no association was found for the RRR-derived unhealthy dietary pattern. Prospective studies are required to further clarify disparities in the association between PCA- and RRR-derived dietary patterns and the risk of GI cancers. **Systematic review registration:** PROSPERO registration no. CRD42022321644.

## INTRODUCTION

Gastrointestinal (GI) cancers refer to cancers in the esophagus, stomach, liver, gallbladder, biliary tract, pancreas, small and large intestine, rectum, and anus.^1^ Recent estimates suggest GI cancers are responsible for 26% of global cancer cases and 25% of cancer-related deaths.^2^ Colorectal cancer (CRC) represents 19.5 of 100 000 cancer cases, followed by, per 100 000, stomach cancer at 11; liver cancer, 9.5; esophageal cancer, 6.3; and pancreatic cancer, 4.9.^3^ In addition to genetics, modifiable lifestyle and environmental factors contribute to the increased risk of GI cancers.^4^^,^^5^

Dietary behavior is a modifiable factor. Consumption of specific food items and nutrients, including high amounts of fruit and vegetables, fiber, and low saturated fatty acids are associated with a lower risk of GI cancers.^6^^,^^7^ Conversely, intake of red and processed meat, sweetened beverages, and alcohol consumption appears to increase the risk of certain GI cancers.^8^ This is mainly based on the association of a single food item or nutrient intake, and the combined effect of overall diet on GI cancer risk has not been thoroughly considered.^9^ This is important because people do not consume food items or nutrients in isolation, and the interaction of food components and nutrients is inevitable.^10^ Given these limitations, examining the whole diet using a posteriori and hybrid data-driven methods may provide more useful information about the association between diet and GI cancer risk.^11^

Principal component analysis (PCA) is a commonly used a posteriori method to derive dietary patterns that maximally explain the variation in the consumed food items without taking into account prior knowledge of the relationship between food/nutrient and health outcomes.^12^ Another dietary pattern analysis method is reduced rank regression (RRR), a hybrid method of dietary pattern analysis that generates dietary patterns by incorporating prior knowledge about the relationship between diet and health outcome, intermediate response variables (eg, fiber, vitamin D, C-reactive protein) and all the food consumed simultaneously in order to identify the food groups that best explain variations in biomarkers of diseases.^11^^,^^13^^,^^14^

The RRR approaches for dietary pattern analyses show associations with broader health outcomes. For example, previous studies have compared the association of dietary patterns derived from PCA and RRR with metabolic health, including overweight/obesity, and cardiovascular diseases, bone mass density,^15^ and diabetes.^16^ Findings suggest that dietary patterns extracted from RRR have stronger associations with health outcomes compared with dietary patterns derived from PCA.^17–19^ The association between PCA- and RRR-derived dietary patterns and cancer risk has also been investigated. Pot et al^20^ compared dietary patterns derived with PCA and RRR and found that only RRR-derived dietary patterns were associated with breast cancer risk, whereas Willemsen et al^21^ found a contrasting finding between PCA- and RRR-derived dietary patterns and risk of lung cancer.

These inconsistent findings highlight the need to clarify which methods of dietary pattern analysis better explain variation of dietary intake, and the mechanism of the diet and GI cancer relationship. To date, to our knowledge, no study has compared the risk differences between dietary patterns derived from a posteriori and hybrid methods and death resulting from GI cancer. Therefore, our aim for this systematic review and meta-analysis was to assess GI cancer risk and mortality associated with dietary patterns derived by a posteriori and hybrid methods.

## METHODS

### Protocol and registration

The protocol was registered in the International Registration of Systematic Reviews (PROSPERO; registration no. CRD42022321644) and can be accessed at https://www.crd.york.ac.uk/prospero/. The Preferred Reporting Item for Systematic Review and Meta-analysis Protocol (PRISMA) 2020^22^ was used to report the findings.

### Study selection and inclusion criteria

The focus for this analysis was on cohort studies that included GI cancer risk or mortality as an outcome together with dietary pattern information. Dietary patterns needed to be constructed by a posteriori or hybrid methods as the exposure variable, reported relative risk (RR) or hazard ratio (HR) with 95% CI, and written in English language to be included in the review. No limitations on publication date were applied.

Cross-sectional and case-control studies, editorial comments, systematic reviews, and meta-analyses were excluded. This exclusion was applied for two main reasons: First, patients might change their dietary habits after a cancer diagnosis. Consequently, assessing the dietary intake of study participants through these designs may not effectively capture their dietary habits before diagnosis, potentially resulting in reverse causation. Second, longitudinal studies offer more robust insights into the temporal relationship between dietary intake and the risk of cancer compared with case-control and cross-sectional studies. A complete summary of eligibility according to PICOS (participants, interventions, comparisons, outcomes, and study design) criteria for inclusion can be found in Table 1.

**Table 1.** PICOS Criteria for Inclusion of Studies

Criterion	Population	Intervention/exposure	Comparator	Outcome	Study design
Inclusion	For GI cancer risk: adults (≥18 y old) who were free of cancers during enrollment in a cohort study and had a noted GI cancer diagnosed during follow up For GI cancer survival: adults (≥18 y old) who were diagnosed with GI cancer during enrollment regardless of their treatment status	A posteriori or hybrid-derived dietary patterns	Greater adherence to specific dietary patterns will be compared with lower adherence	GI cancer risk and/or death	Cohort studies
Exclusion	<18 y old	A priori dietary patterns or single food items and nutrients patterns	None	GI adenoma (precancerous lesions) as an outcome of interest	Cross-sectional and case-control studies, editorial comments, systematic reviews, and meta-analyses

Abbreviation: GI, gastrointestinal.

### Search strategy

Initially, we searched for articles in Ovid Medline, SCOPUS, Cochrane Library, CINHAL, PsycINFO, ProQuest, and Web of Sciences electronic databases for any prospective studies published up to May 26, 2022, and last updated on June 23, 2023. The search terms were developed using free-text words and Medical Subject Headings, including search terms such as GI cancers, dietary patterns, and a posteriori and hybrid dietary pattern analysis methods. Subsequently, studies were searched in Google Scholar and the reference lists of the retrieved studies and previous systematic reviews were checked for both grey and published literature. No further prospective studies were identified. The detailed search strategy for each database can be found in Table S1.

All studies retrieved through the search strategy were imported to Covidence. After duplication removal, studies were screened in duplicate by 2 reviewers (Z.A. and T.C.M.) using title and abstract to select studies fulfilling the inclusion criteria. The full-text review was conducted independently by 2 authors (Z.A. and T.C.M.), and any conflicts were resolved by a third author (M.M.W.).

### Data extraction

A data extraction sheet was prepared and tested on a subsample of studies for necessary correction before starting the extraction of all included studies for the final analysis. Two authors (Z.A. and T.C.M.) extracted the data independently. Any disagreement during extraction between the two authors was resolved via discussion, which was supported by a third author (M.M.W.). A detailed summary of data extracted for each study is provided in Table S2. The risk estimates of overall GI cancers, and by specific sites (namely, stomach, esophagus, oral, CRC [proximal colon, distal colon, rectum, or all colon], liver, pancreas, and gastric), were extracted where possible. When studies reported several risk and mortality estimates with different degrees of adjustment, the estimate adjusted for the most covariates was used.

### Quality assessment

Full-text studies were assessed for risk of bias using the Newcastle-Ottawa Scale (NOS).^23^ Briefly, the NOS has 9 items categorized into 3 domains, which are assessed with a star system. Each item included the following domains: selection (1–4 stars): representativeness of the exposed cohort, selection of the nonexposed cohort, ascertainment of exposure, demonstration that outcome of interest was not present at the start of the study; comparability (1–2 stars): comparability based on the control group for age, sex, and family history of GI cancers and additional adjustment during analysis for at least one of the following: body mass index (BMI), smoking, alcohol drinking, and energy intake; Outcome (1–3 stars): assessment of outcome, a 10- and 5-year follow-up long enough for GI cancer risk and mortality, respectively, to occur, and at least 80% follow-up of cohorts at the end of the study period (maximum of 20% lost to follow up).

One star was given for the selection and outcomes domains and a maximum of two stars for the comparison domain; thus, the NOS has a maximum score of 9. Accordingly, a score ≥7 indicates high quality, 4-6 indicates moderate quality, and <4 indicates low quality.^24^ Quality assessment was completed by two reviewers (Z.A. and T.C.M.), and disagreements were resolved by a third author (M.M.W.).

### Dietary pattern identification

Because of the subjective naming of dietary patterns in the original studies (eg, “healthy,” “unhealthy,” “prudent,” “drinker,” and “vegetables”), together with the heterogeneity of these dietary patterns between studies, factor loading was used to determine which dietary patterns are classified as “healthy” and “unhealthy.” Specifically, the weighted average factor loading was calculated for each dietary pattern to improve the homogeneity among the included studies. Dietary patterns were named on the basis of the factor loading of the majority of food groups or items.

To determine which foods were linked to a higher or lower risk of GI cancers, the World Cancer Research Fund (WCRF)/America Institute for Cancer Research (AICR) dietary guidelines were used to list food items.^8^ In the WCRF/AICR guidelines, food groups or items identified as being associated with a convincing increased risk, probably increased risk, and limited suggestive increased risk for development of GI cancers were categorized as being associated with increased risk. Similarly, food groups or items classified as being associated with a convincing decreased risk, probably decreased risk, and limited suggestive decreased risk were categorized as being associated with a decreased risk for development of GI cancers.

Fruits, vegetables, legumes, whole grains, milk and milk products, coffee, and fish were classified as food groups or items that decreased the risk of GI cancers. In contrast, red and processed meat, alcohol, refined grains, processed and fast foods, salted fish, sugar-sweetened drinks, and processed nonstarchy vegetables were classified as those increasing the risk.

A weighted average of factor loadings was calculated for each dietary pattern based on factor loadings and number of food groups. To determine the weighted average, an average factor loading of each dietary pattern was taken first. Then, this average factor loading was multiplied by the number of food groups in the dietary patterns that were used for the computation of average factor loadings. In the case of cross-loading, the same procedure was followed but separately for negative and positive factor loadings. Then, the weighted average factor loadings were summed, resulting in either negative or positive weighted average factor loading. If the difference was more than the absolute value of 0.3, the factor was considered as contributing significantly to the dietary pattern^25^^,^^26^ and was included in the meta-analyses. The difference was estimated between the weighted average factor loading of decreased risk and increased risk. When an article’s authors reported a separate factor loading for men and women, a separate weighted average factor loading was calculated. The names of food items listed under each dietary pattern were quantitatively checked if factor loadings were not reported.

Because there was no factor loading reported for RRR-derived dietary patterns, the weighted average factor loading was not calculated. Therefore, dietary patterns were categorized as healthy or unhealthy dietary patterns on the basis of the characteristics of the food items reported under each dietary pattern (Table S3).

Finally, a healthy dietary pattern is one that encompasses all the foods and beverages people regularly consume, recognizing that the various components of this pattern work in synergy to decrease the risk of GI cancers.^27^ This healthy pattern is characterized by greater intake of fruits, vegetables, legumes, whole grains, milk and milk products, coffee, and fish. In contrast, an unhealthy dietary pattern elevates the risk of GI cancers and is characterized by a higher intake of red and processed meat, alcohol, refined grains, processed and fast foods, salted fish, sugar-sweetened drinks, and processed nonstarchy vegetables.

### Data analysis

When studies reported the association of dietary patterns with GI cancer risk for men and women separately, the study was considered, for meta-analytic purposes, as two independent studies. In addition, the original studies may have more than one healthy or unhealthy dietary patterns and, consequently, may have been counted multiple times. Where this occurred, this is clearly reported.

In the present meta-analyses, studies that used cluster analysis (CA) to determine dietary patterns have been excluded for methodological reasons. Unlike PCA and RRR, CA creates mutually exclusive dietary patterns, making it challenging to combine the results in a meta-analysis. Furthermore, in CA, the reference categories for regression are independent and distinct dietary patterns, dietary patterns vary across studies, and estimates do not compare low vs high adherence to a particular dietary pattern. In contrast, PCA- and RRR-derived factor scores were converted to tertiles, quartiles, or quintiles. All authors consistently used the first tertile, first quartile, or first quintile as their reference group, making meta-analysis possible.

The *meta* and *metafor* packages^28^ in R, version 4.13, and RStudio^29^ were used to pool maximally adjusted estimates. For this study, RR and HR were treated as the same and reported as RR.^30^ A random-effects model was used to calculate the pooled RR and 95% CI, comparing the highest intake of foods associated with healthy and unhealthy dietary patterns with the lowest intake category (reference group). The DerSimonian-Laird method was used to calculate between-study heterogeneity variance, *τ*^2^.^31^

The degree of heterogeneity among included studies was checked quantitatively using the *I^2^* statistic. The percentage of *I^2^* values of 0%-40%, 30%-60%, 50%-90%, and 75%-100% indicates low, moderate, substantial, and considerable high heterogeneity, respectively.^32^ Similarly, small-study effects and publication bias were evaluated using the visual funnel plot test and Egger’s and Begg’s statistics^33^ in the random-effect model, and a 0.05 level of significance was used to detect asymmetry.

Finally, a subgroup analysis was conducted to assess the association between dietary patterns and specific types of GI cancers, and by sex of study participants. Subgroup analysis was considered for dietary patterns with >10 estimates. In addition, the presence of statistically significant subgroup difference (*P* < .05), degree of heterogeneity, unequal distribution of covariates among groups, and clinical significance to report the findings were considered.^34^

### Grading the level of evidence

The certainty of evidence from the meta-analyses was assessed using the Grading of Recommendations Assessment, Development and Evaluation (GRADE) criteria.^35^ Based on the risk of bias, inconsistency (heterogeneity), indirectness, and publication bias, the certainty of evidence was classified as very low, low, moderate, or high.^35^ According to GRADE principles, the level of evidence from an observational study is classified as low.^36^

## RESULTS

### Search results

**Figure 1.**
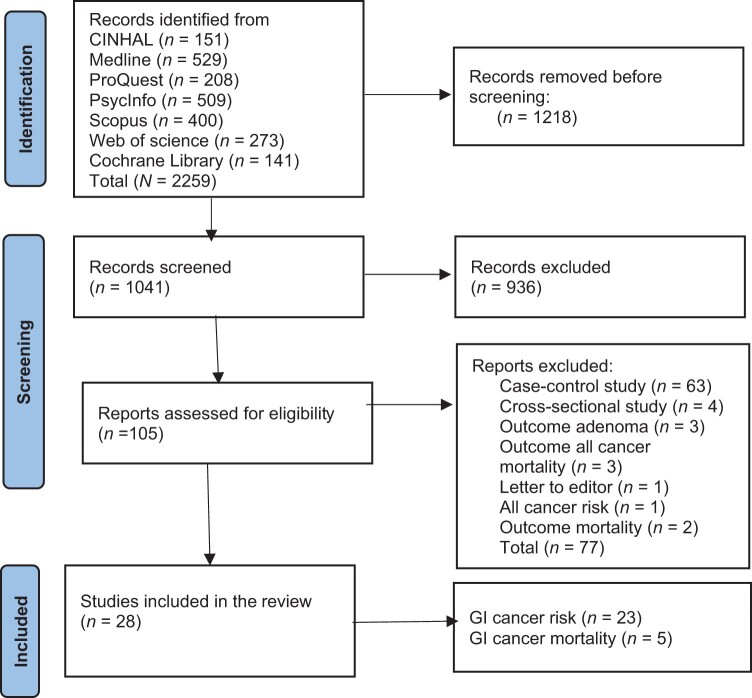
PRISMA Flow Diagram Showing Study Literature Search and Inclusion in Systematic Review and Meta-Analysis

A total of 2259 articles were identified in the initial search, and 1218 were excluded during the title and abstract screening (Figure 1^21^^,^^26^^,^^37–62^). Of 105 articles considered for full-text evaluation, 77 were excluded because they did not fulfill the inclusion criteria (Table S4). Therefore, this review included a total of 28 articles that were published from 2001 to 2023 with a combined total of 1 718 542 participants.

### Characteristics of included studies

Studies in this review were conducted in different countries, based on different methods of dietary analysis, and considered different types of GI cancers. Eight of the 28 studies were conducted in the United States,^43–45^^,^^52^^,^^56^^,^^57^^,^^59^^,^^60^ 5 in Japan,^42^^,^^46–49^^,^^63^^,^^64^ 2 in Canada,^21^^,^^54^, 2 in China,^37^^,^^53^ and 1 each in Korea,^38^ Iceland,^40^ Sweden,^41^ France,^50^ Taiwan,^51^ Norway,^58^ Finland,^61^ the United Kingdom,^26^ and Singapore.^62^ There were 6 studies conducted with only female participants^41^^,^^50^^,^^52^^,^^56^^,^^58^^,^^60^ and 3 studies with only male participants.^46^^,^^61^^,^^63^ Of 28 studies, 22 studies used PCA,^26^^,^^37–53^^,^^63^^,^^64^ 2 used CA,^57^^,^^58^ 1 combined PCA and RRR,^21^ 1 combined PCA and CA,^54^ and 2 used RRR to derive dietary patterns.^55^^,^^56^ Of the articles included, 23 were about studies related to the risk of GI cancers. Of these studies, 14 focused on CRC, 2 on the liver, 2 on general GI, 3 on the stomach, and 2 on the pancreas. Most of the studies included age, sex, BMI, family history, and energy intake as confounding variables within multivariable analyses (Table 2^21^^,^^26^^,^^37–62^).

**Table 2.** A Summary of Studies of Dietary Patterns and GI Cancer Risk

Author (year)	Country	Sample size	Sex	Age (y)	Dietary data collection period	Types of cancer	Analysis methods for the association between dietary patterns and cancer risk	Variable used for adjustment
Zhang et al (2013)^37^	China	132 837	Both	40-74	Baseline and after 2-3 y	Liver	Cox proportional hazard regression model	Sex, age, BMI, total energy intake, family income level, education level, family history of liver cancer in first-degree relatives, history of: chronic viral hepatitis, chronic liver disease or cirrhosis, diabetes, cholelithiasis or cholecystectomy, and vitamin C and E and multivitamin supplement use, and mutual adjustment for 3 dietary patterns
Wirfalt et al (2009)^57^	United States	492 306	Both	50-71	1995-1996 (baseline to assess dietary intake over the past 1 y)	CRC	Cox proportional hazards regression	Age, BMI, education, ethnicity, smoking, leisure time physical activity, and total energy (continuous), MHT
Willemsen et al (2022)^21^	Canada	26 462	Both	35-69	Baseline to assess dietary intake over the preceding 12 mo	Colon	Cox proportional hazard regression models	Age, sex, BMI, energy intake, smoking status, physical activity
Wie et al (2017)^38^	Korea	8024	Both	>20	Baseline using 3-d food record	GI	Cox proportional hazards regression model	Age, sex, energy intake, BMI, physical activity, smoking, alcohol use, income, education, and marital status
Thordardottir et al (2022)^40^	Iceland	5078	Both	≥67	During adolescence period (ages 14-19 y), in midlife (40-50 y), and at study entry (67 y or older) The baseline was used for this analysis.	CRC	Cox proportional hazard regression models	Age, sex, BMI, educational status, family history of CRC, and physical activity
Terry et al (2001)^41^	Sweden	61 463	Female	40-74	Baseline to assess dietary exposure for the past 6 mo	CRC	Cox proportional hazards model	Age, energy intake, BMI, and educational status
Shin et al (2018)^42^	Japan	93 062	Both	40-69	5 y after enrollment to assess food intake over the previous year	CRC	Cox proportional hazard model	Age, public health center area, BMI, smoking status, total physical activity, menopause status, log-transformed total energy intake, and use of exogenous female hormones
Nothlings et al (2008)^55^	Multiple countries^a^	183 513	Both	45-75	Baseline to assess dietary intake during the previous year	Pancreatic	Cox proportional hazard models	Sex, race and ethnicity, follow-up time, BMI, prevalent diabetes mellitus, family history of pancreatic cancer, age at cohort entry, energy intake (logarithmically transformed), pack-years of smoking, and smoking status
Michaud et al (2005)^43^	United States	124 672	Both	30-75	1984 (for women) and 1986 (for men) asked to report their average frequency of intake over the previous year	Pancreatic	Cox proportional hazards regression	Age, pack-years of smoking, BMI, physical activity, history of diabetes mellitus, caloric intake, height, and multivitamin use
Mehta et al (2017)^45^	United States	137 217	Both	30-75	1980, 1984, 1986, 1990, 1994, 1998, 2002, 2006, and 2010 for the Nurses’ Health Study, and 1986, 1990, 1994, 1998, 2002, 2006, and 2010 for the Health Professionals Follow-up Study	CRC	Cox proportional hazards regression models	Age, calendar y, and sex, and adjusted for total caloric intake, family history of CRC in any first-degree relative, history of the previous endoscopy, pack-years of smoking, BMI, physical activity, and regular aspirin or nonsteroidal anti-inflammatory drug use
Masaki et al (2003)^46^	Japan	5644	Male	40-69	1988 (baseline) to assess the past 2 or 3 y	Gastric or stomach cancer	Cox proportional hazard regression model	Age, BMI, education, history of peptic ulcer, family history of stomach cancer, status of cigarette smoking and alcohol drinking
Kumagai et al (2014)^47^	Japan	44 097	Both	40-79	Baseline (1994)	CRC	Cox proportional hazard regression model	Age, sex, BMI, smoking status, walking duration, education, total energy intake, and family history of CRC
Kim et al (2004)^49^	Japan	42 112	Both	40-59	Baseline	Gastric cancer	Cox proportional hazard regression models	Age, BMI, energy intake, education level, family history of gastric cancer, smoking status (for men), and alcohol drinking (for men)
Kim et al (2005)^48^	Japan	42 112	Both	40-59	Baseline	CRC	Cox proportional hazard regression model	Age, BMI, study area (for the healthy and Western dietary pattern), energy intake, education level, physical activity, family history of CRC, smoking status (for men), and alcohol consumption (for men)
Kesse et al (2006)^50^	France	68 442	Female	40-65	1993-1995	CRC	Cox proportional hazard regression models	Daily energy intake,BMI, physical activity level, tobacco status, family history of CRC, and high education level
Hsiung et al (2016)^51^	Taiwan	1211	Both	16-80	Dietary data were collected at 3 age bands: <16 y, 16–30 y, and >30 y	Gastric cancer	Cox proportional hazard regression models	The extracted results are from a univariable model.
Fung et al (2012)^56^	United States	66 714	Female	30-55	1986, 1990, 1994, 1998, and 2002	CRC	Cox proportional hazard models	Age, energy intake, pack-years of smoking, multivitamin use, family history, aspirin use, history of polyps, colonoscopy screening, alcohol intake, physical activity, BMI
Fung et al (2003)^60^	United States	76 399	Female	30-55	Baseline (1984)	Colon	Cox proportional hazard regression models	Age, time, smoking, BMI, aspirin use, total energy, physical activities, alcohol intake, multivitamin use, missing food frequency questionnaire, and family history
Flood et al (2008)^59^	United States	492 382	Both	>50	1995-1996 (baseline)	CRC	Cox proportional hazards regression	Ethnicity, tobacco use, physical activity, BMI, education, family history of colon cancer, regular nonsteroidal anti-inflammatory drug use, and, in the case of women, use of menopausal hormones
Engeset et al (2009)^58^	Norway	34 471	Female	47.6 (mean age)	1991/92-1998	GI cancer	Cox regression model	Age, smoking, energy, and education
Dixon et al (2004)^61^	Finland	29 133	Male	50-69	Baseline to assess dietary exposure in the previous y	Colon and rectal	Cox proportional hazards regression model	Age, ATBC treatment group, BMI, education, smoking (number of cigarettes/d, years smoked), occupational activity, and energy intake
Butler et al (2008)^62^	Singapore	61 321	Both	45-74	Baseline to assess usual diet over the past y	CRC	Proportional hazards regression model	Age, sex, dialect group, interview year, diabetes at baseline, smoking history, BMI, alcohol intake, education, physical activity, first-degree relative diagnosed with CRC, and total daily energy intake
Guo et al (2022)^26^	United Kingdom	372 492	Both	40-70	Baseline	Liver	Cox proportional hazards regression model	Age, sex, race, education level, Townsend deprivation index (quartiles), drinking status, smoking status, exercise, BMI, and diabetes

a Study conducted in different countries, including Norway, Sweden, Denmark, the United Kingdom, the Netherlands, Germany, France, Italy, Spain, and Greece.

Abbreviations: ATBC, alpha-tocopherol beta-carotene cancer prevention study; BMI, body mass index; CRC, colorectal cancer; GI, gastrointestinal; MHT, menopausal hormone therapy.

### Dietary patterns

In most cases, the dietary patterns prepared on the basis of factor loading were the same as the dietary patterns reported by the articles’ authors. The exceptions to this were 1 unhealthy dietary pattern reported by authors, which was analyzed as a healthy dietary pattern^42^ according to the factor loading approach, and 1 unhealthy type of dietary pattern that was classified as neither a healthy nor unhealthy dietary pattern.^46^ Three traditional dietary patterns^42^^,^^48^^,^^49^ reported by original studies were changed to healthy dietary patterns for women only (Table S3). However, 2 articles^39^^,^^52^ did not include factor loading, and they simply reported 2 dietary patterns, as healthy and unhealthy, that aligned with the criteria used in this study and thus were directly included in the pooled estimates. Finally, a total of 42 healthy and 29 unhealthy PCA-derived dietary patterns were used to estimate GI cancer risk.

### PCA-derived dietary patterns and GI cancer risk

All studies used a food frequency questionnaire (FFQ) to assess dietary exposure, except 1, which used dietary records for 3 days.^38^ The number of food items included in the FFQ ranged from 31 to 276. However, 2 studies did not provide information on the number of food items included in the FFQ.^40^^,^^49^ The frequency of dietary assessments during the follow-up period ranged from 1 to 9 times, and the authors used the first (baseline) survey to assess the association between dietary intake and GI cancer risk. Twenty-three of the included studies used validated FFQs for dietary assessment, but 3 studies lacked information on the validity and reproducibility of the dietary assessment (Table 3^21^^,^^26^^,^^37–62^). CRC risk was the most commonly studied end point, followed by stomach cancer, and a combined GI cancers risk.

**Table 3.** PCA-Derived Dietary Patterns and GI Cancer Risk

Author (year)	Country	No. of food items included	Validation of dietary assessment tool	Reproducibility of a dietary assessment tool	Use of loading >0.3 scoring criteria
Zhang et al (2013)^37^	China	SWHS, *n* = 77; and SMHS, *n* = 81	Yes	Yes	Not mentioned
Wirfalt et al (2009)^57^	United States	204	Yes	Yes	Yes
Willemsen et al (2022)^21^	Canada	124	Yes	Yes	Yes
Wie et al (2017)^38^	Korea	Not mentioned	Yes	Yes	Not mentioned
Van Blarigan et al (2020)^39^	United States	130	Yes	Yes	Not mentioned
Thordardottir et al (2022)^40^	Iceland	Not mentioned	No	No	Yes
Terry et al (2001)^41^	Sweden	67	Not mentioned	Not mentioned	No
Shin et al (2018)^42^	Japan	138	Yes	Yes	Not mentioned
Sharma et al (2018)^54^	Canada	169	Yes	Not mentioned	Not mentioned
Nothlings et al (2008)^55^	Multiple countries^a^	51	Yes	Yes	Yes
Michaud et al (2005)^43^	United States	130	Yes	Yes	No
Meyerhardt et al (2007)^44^	United States	131	Yes	Yes	Not mentioned
Mehta et al (2017)^45^	United States	131	Yes	Yes	Yes
Masaki et al (2003)^46^	Japan	33	Yes	Yes	No
Kumagai et al (2014)^47^	Japan	44	Yes	Yes	No
Kim et al (2004)^49^	Japan	Not mentioned	Yes	Yes	No
Kim et al (2005)^48^	Japan		Yes	Yes	No
Kesse et al (2006)^50^	France	208	Yes	Yes	No
Hsiung et al (2016)^51^	Taiwan	31	Not mentioned	Not mentioned	Yes
Fung et al (2014)^52^	United States	130	Yes	Not mentioned	Not mentioned
Fung et al (2012)^56^	United States	116	Yes	Yes	Yes
Fung et al (2003)^60^	United States	116	Yes	Yes	Not mentioned
Flood et al (2008)^59^	United States	204	Yes	Yes	Not mentioned
Engeset et al (2009)^58^	Norway	86	Yes	Yes	Yes
Dixon et al (2004)^61^	Finland	276	Yes	Not mentioned	Yes
Butler et al (2008)^62^	Singapore	165	Yes	Yes	Yes
GUO et al (2022)^26^	United Kingdom	15	Yes	Yes	Yes
Zhao et al (2022)^53^	China	14	Yes	Yes	Not mentioned

a Study conducted in different countries including Norway, Sweden, Denmark, the United Kingdom, the Nether-lands, Germany, France, Italy, Spain, and Greece.

Abbreviations: SWHS, Shanghai Women's Health Study; SMHS, Shanghai Men's Health Study.

**Figure 2.**
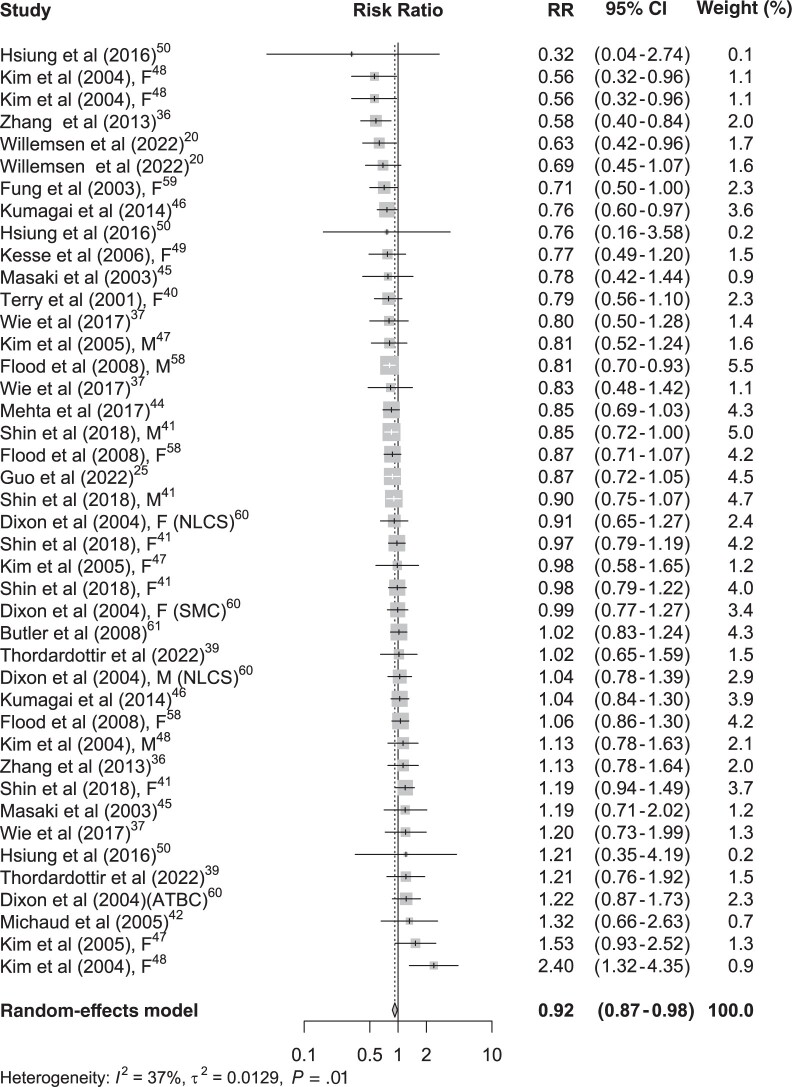
Forest Plot of the Association between Principal Component Analysis–Derived Healthy Dietary Patterns and Gastrointestinal Cancers. Abbreviations: ATBC, alpha-tocopherol beta-carotene cancer prevention study; F, female; M, male, NLCS, Netherlands cohort study on diet and cancer; SMC, Swedish mammography cohort.

**Figure 3.**
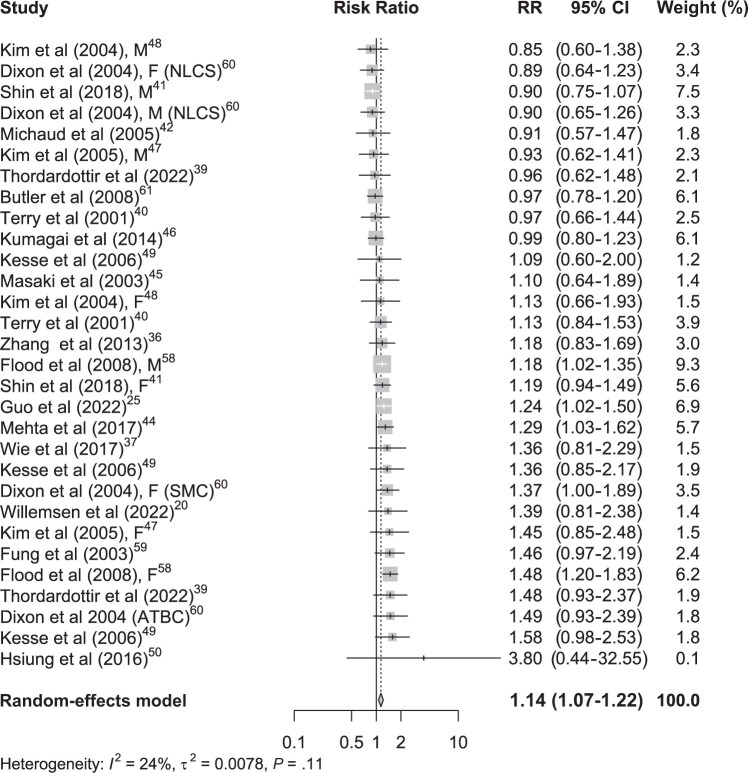
Forest Plot of the Association between Unhealthy Dietary Patterns and Gastrointestinal Cancers. Abbreviations: ATBC, alpha-tocopherol beta-carotene cancer prevention study; F, female; M, male, NLCS, Netherlands cohort study on diet and cancer; SMC, Swedish mammography cohort.

Among the studies that used PCA to derive dietary patterns, 42 estimates of healthy dietary patterns and GI cancer risk were extracted. Of these, 6 estimates had a significant protective association with GI cancer risk (Figure 2^21^^,^^26^^,^^37^^,^^38^^,^^40–43^^,^^45–51^^,^^59–62^). The remaining 36 did not show a statistically significant difference between higher and lower intake of foods included in healthy dietary patterns and GI cancer risk. A total of 29 PCA-derived unhealthy dietary patterns and GI cancer risk estimates were used in this meta-analysis. Only 5 had a positive statistical association with the risk of GI cancers. None of the estimates had a significant inverse association with the risk of GI cancers (Figure 3^21^^,^^26^^,^^37^^,^^38^^,^^40–43^^,^^45–50^^,^^59–62^).

### RRR-derived dietary patterns and GI cancer risk

Three studies used RRR to determine dietary patterns. The studies used different response variables to derive dietary patterns (Table 4^21^^,^^55^^,^^56^). A study conducted by Willemsen et al^21^ considered dietary fiber, vitamin D, fructose, and discretionary fat as response variables. The authors selected the response variable based on the availability of evidence between the response variable and cancer risk and, accordingly, dietary fiber and vitamin D are supposed to decrease the risk of CRC, whereas, fructose and discretionary fat increase CRC risk. However, the authors found dietary patterns derived based on discretionary fat had a protective effect against CRC.^21^ Fung et al^56^ showed that RRR-derived fasting plasma C-peptide dietary patterns were positively associated with CRC and colon cancer risk, whereas no significant association was found for rectal cancer. Finally, Nothlings et al^55^ used quercetin, kaempferol, and myricetin to derive dietary patterns that are associated with pancreatic cancer.

**Table 4.** Summary of Reduced Ranked Regression–Derived Dietary Patterns and Cancer Risk

Author (year)	Country	No. of food items included	Validation of dietary assessment tool	Reproducibility of a dietary assessment tool	Intermediate response variables	Use of loading >0.3 scoring criteria	Type of cancer studied	**Dietary patterns and risk association (HR** **with 95% CI)**
Willemsen et al (2022)^21^	Canada	124	Yes	Yes	Dietary fiber, vitamin D, fructose, and discretionary fats	Yes	Colon	Dietary fiber: HR, 0.54 (0.31-0.92) Vitamin D: HR, 1.02 (0.66-1.57) Fructose: HR, 1.09(0.75-1.6) Discretionary fats: HR, 0.52 (0.33-0.82)
Nothlings et al (2008)^55^	Multiple countries^a^	51	Yes	Yes	Quercetin, kaempferol, and myricetin	Not mentioned	Pancreatic cancer	Quercetin, kaempferol, and myricetin dietary patterns: HR, 0.88 (0.67-1.15)
Fung et al (2012)^56^	United States	116	Yes	Yes	Fasting plasma C-peptide	Not mentioned	CRC	CRC; C-peptide dietary pattern score: HR, 1.29 (1.05-1.58) Rectal; C-peptide dietary pattern score: HR, 1.07(0.69-1.65) Colon; C-peptide dietary pattern score: HR, 1.35(1.07-1.7)

a Study conducted in different countries, including Norway, Sweden, Denmark, the United Kingdom, the Netherlands, Germany, France, Italy, Spain, and Greece.

Abbreviations: CRC, colorectal cancer; HR, hazard ratio.

### PCA-derived dietary patterns and GI cancer–caused death

A total of 5 studies examined the relationship between dietary patterns and GI cancer survival. All studies used the Cox proportional hazard model to assess the relationship between dietary patterns and mortality from GI cancers. Four of the 5 studies reported CRC^39^^,^^44^^,^^52^^,^^54^ and 1 esophageal cancer^53^ survivorship. In addition, each study used different timing of dietary data collection. In their studies, Sharma et al^54^ and Zhao et al^53^ collected dietary data before cancer diagnosis; in 3 studies,^39^^,^^44^^,^^52^ researchers collected dietary data after diagnosis. As a result, meta-analysis was not performed for PCA-derived dietary patterns and GI cancer survivorship.

A variety of dietary patterns and different results were identified from the included cohort studies. Studies conducted by Van Blarigan et al,^39^ Fung et al,^52^ and Zhao et al^53^ showed that a prudent pattern had a reduced risk of death resulting from CRC and esophageal cancer. All healthy dietary patterns were characterized by a higher intake of vegetables, legumes, and fruits.

On the other hand, Meyerhardt et al^44^ showed that an unhealthy dietary pattern characterized by refined grains, processed and red meats, desserts, high-fat dairy products, and French fries was associated with an increased risk of death from colon cancer (HR, 2.32; 95% CI, 1.36-3.96). Similarly, unhealthy dietary patterns reported by Sharma et al,^54^ Fung et al,^52^ and Zhao et al^53^ were positively associated with increased risk of dying from CRC and esophageal cancer, but they found no statistically significant association (Table 5^39^^,^^44^^,^^52–54^).

**Table 5.** Summary of Studies on Dietary Patterns and GI Cancer Mortality

Author (year)	Country	Median follow-up time	Sample size	Sex	Age (y)	Dietary data collection period	Types of cancer	Multivariate adjusted HR (95% CI)	Variable used for adjustment
Van Blarigan et al (2020)^39^	United States	73 (IQR, 64-87) mo	1284	Both	>18	Within 4 wk after treatment initiation to capture the past 3 mo of dietary intake	CRC	Prudent: 0.83 (0.66-1.04) Western; 0.85 (0.65-1.10)	Age, sex, energy intake (kcal/d), race/ethnicity; performance status; protocol chemotherapy; primary tumor unresected; diabetes; treatment arm; KRAS status; tumor sidedness; weight change in previous 6 mo; BMI, and physical activity
Sharma et al (2018)^54^	Canada	6.27 ± 1.98 y	532	Both	20-75	Retrospectively a year before the diagnosis	CRC	Prudent: vegetable: 1.03 (0.61-1.75) Processed meat: 1.53 (0.85-2.27) High sugar: 1;27 (0.72- 2.23)	Energy, stage of cancer, sex, age, marital status, tumor location, screening history, intake of alcohol, radiation, chemotherapy status, microsatellite instability status
Meyerhardt et al (2007)^44^	United States	5.3 years	1009	Both	No report	Middle of their adjuvant chemo-therapy course and approximately 6 mo after the completion of adjuvant therapy to assess the past 3 mo of dietary intake	Colon cancer	Prudent: 1.32 (0.86-2,04) Western: 2.32 (1.36-3.96)	Sex; age; depth of invasion through the bowel wall (T1-2 vs T3-4); number of positive lymph nodes (1-3 vs 4); presence of clinical perforation at the time of surgery; presence of bowel obstruction at the time of surgery; baseline performance status (0 vs 1-2); treatment group; weight change between first and second questionnaire; and time-varying BMI, physical activity level, and total calories
Fung et al (2014)^52^	United States	11.2 years	1201	Female	30-55	6 mo after diagnosis	CRC	Prudent: 0.67 (0.37-1.22) Western: 1,66 (0.85-3.23)	Age, physical activity, BMI, weight change, cancer grade, chemotherapy, smoking status, energy intake, colon or rectal cancer, stage disease, and date of CRC diagnosis
Zhao et al (2023)^53^	China	No data	855	Both	40-70	Dietary intake over past 5 y before diagnosis	Esophageal squamous cell carcinoma	Pattern 1: 0.94 (0.77-1.15) Pattern 3: 0.90 (0.66-1.24) Pattern 2: 1.21 (0.98-1.51)	Age, sex, marital status, educational level, BMI, family cancer history, weekly alcohol intake, cigarette smoking (pack-year), energy intake (MJ/d), tumor stage, tumor location, anticancer treatment effect, treatment approach, diet quality on discharge, sleep quality on discharge, and daily living activity on discharge

Abbreviation: CRC, colorectal cancer; HR, hazard ratio.

Altogether, the findings indicate healthy dietary patterns may reduce the risk of death dur to GI cancer, whereas unhealthy dietary patterns may increase the risk.

### Pooled estimates of PCA dietary patterns and GI cancer risk

Overall, compared with the lowest intakes of foods in a healthy dietary pattern, the highest intake those foods reduced the risk of GI cancers by 8% (pooled RR, 0.92; 95% CI, 0.87-0.98), with a low degree of heterogeneity (*I*^2^ = 37%; *P* = .01) (Figure 2^21^^,^^26^^,^^37^^,^^38^^,^^40–43^^,^^45–51^^,^^59–62^).

In contrast, as shown in Figure 3,^21^^,^^26^^,^^37^^,^^38^^,^^40–43^^,^^45–50^^,^^59–62^ the highest intake of foods in unhealthy dietary patterns was positively associated with an increased risk of GI cancers by 14% (pooled RR, 1.14; 95% CI, 1.07-1.22), with a low degree of heterogeneity (*I*^2^ = 24%; *P* = .11).

### Pooled estimates of RRR-derived dietary patterns and GI cancer risk

**Figure 4.**
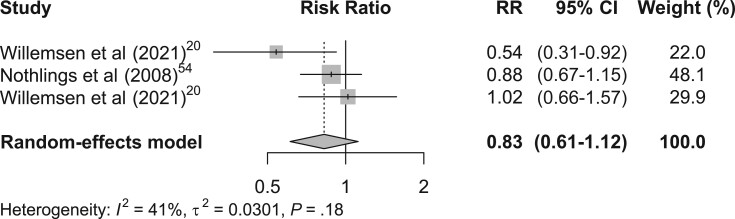
Forest Plots of the Association between RRR-Derived Healthy Dietary Patterns and Gastrointestinal Cancer Risk.

**Figure 5.**
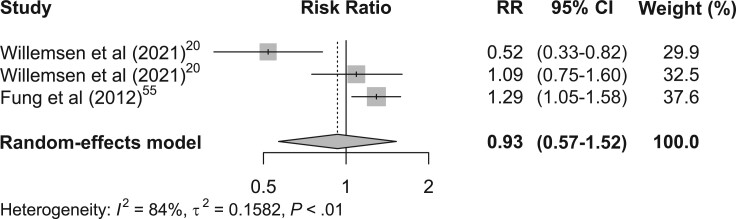
Forest Plots of the Association between RRR-Derived Unhealthy Dietary Patterns and Gastrointestinal Cancers Risk.

A pooled estimate of RRR-derived healthy dietary pattern and GI cancers risk showed that a healthy dietary pattern may reduce the risk of GI cancers (RR, 0.83; 95% CI, 0.61-1.12), with moderate heterogeneity (*I*^2^ = 41.4; *P* = .18) (Figure 4^21^^,^^55^). Similarly, when RRR-derived unhealthy dietary patterns were pooled together, the results showed no significantly increased risk of GI cancers (Figure 5^21^^,^^56^), but there was significant heterogeneity (*I*^2^ = 84%; *P* < 0.01).

### Subgroup analysis

The absence of a statistically significant subgroup effect for sex (*P* = .71 and .08 for healthy and unhealthy dietary patterns, respectively) suggested that the sex of study participants does not modify the effect of the PCA-derived dietary patterns on GI cancer risk. In men, although fewer studies were included, a higher intake of foods that are part of a healthy dietary pattern was associated with a 9% reduced risk of GI cancers (RR, 0.91; 95% CI, 0.83-0.99), with low heterogeneity (*I*^2^ = 16%; *P* = .30; Figure S1). In contrast, greater adherence to unhealthy dietary patterns was associated with a 25% increased risk of GI cancers among women (RR, 1.25; 95% CI, 1.13-1.38), with low heterogeneity (*I*^2^ = 4%; *P* = .41; Figure S2).

Subgroup analysis based on specific types of GI cancers showed no statistically significant subgroup effect (*P* = .84 and .60 for healthy and unhealthy dietary patterns, respectively). Fewer studies of liver, pancreas, and stomach cancers were included, which may limit the ability to determine meaningful subgroup differences. However, higher intake of foods associated with a PCA-derived healthy dietary pattern was associated with a reduced risk of CRC (RR, 0.92; 95% CI, 0.87-0.98), with low heterogeneity (*I*^2^ = 28%; *P* = .09; Figure S3), and higher intake of foods from a PCA-derived unhealthy dietary pattern was associated with increased risk of CRC by 14% (RR, 1.14; 95% CI, 1.05-1.24), with a low degree of heterogeneity (*I*^2^ = 37%; *P* = .04) and liver cancer by 23% (RR, 1.23; 95% CI, 1.03-1.45), with low degree of heterogeneity (*I*^2^ = 0%; *P* = .81; Figure S4).

When the analysis was restricted to colon and rectum cancers only, there was no statistically significant subgroup effect (*P* = .71 and 0.77 for PCA-derived healthy and unhealthy dietary patterns, respectively). However, PCA-derived healthy dietary pattern was associated with a 10% decreased risk of colon cancer (RR, 0.90; 95% CI, 0.82-0.98), with a low degree of heterogeneity (*I*^2^ = 32.1%; *P* = .09; Figures S5 and S6).

### Publication bias

The visual funnel plots for both dietary patterns were symmetrical (Figures S7 and S8). In addition, Egger's test showed no evidence of publication bias for healthy (*P* = .77) and unhealthy (*P* = .31) dietary patterns. Similarly, for RRR-derived dietary patterns, the funnel plot is slightly asymmetrical, but Egger's test showed no evidence of significant publication bias (*P* = .64 and *P* = .34 for healthy and unhealthy dietary patterns, respectively (Figure S9 and S10).

### Quality assessment

The overall quality assessment of included studies was moderate to high. Twenty studies had a score of ≥7, and 8 studies had a score of 6. All studies included cancer-free individuals to estimate the association between dietary patterns and GI cancer risk. Sixteen studies followed the participants for >10 years to assess the outcome. One study used weighted food record methods to measure dietary exposure; the other studies used self-reported or structured interview FFQs. Sixteen studies were adjusted for age, sex, and family history. All studies were additionally adjusted for BMI and/or energy intake and/or smoking and/or alcohol drinking. The detailed quality assessment score is provided in Table 6.^21^^,^^26^^,^^37–62^

**Table 6.** Quality Assessment of Included Studies^a^

Author (year)	Selection				Comparability		Outcome			Total score
	1	2	3	4	1A	1B	1	2	3	
Zhang et al (2013)^37^	*		*	*	*	*	*		*	7
Wirfalt et al (2009)^57^	*		*	*		*	*		*	6
Willemsen et al (2022)^21^	*		*	*		*	*	*	*	7
Wie et al (2017)^38^	*		*	*		*	*		*	6
Van Blarigan et al (2020)^39^	*		*	*		*	*	*	*	7
Thordardottir et al (2022)^40^	*		*	*	*	*	*			6
Terry et al (2001)^41^	*		*	*		*	*		*	6
Shin et al (2018)^42^	*		*	*		*	*	*	*	7
Sharma et al (2018)^54^	*		*	*		*	*	*		6
Nothlings et al (2008)^55^	*		*	*	*	*	*	*	*	8
Michaud et al (2005)^43^			*	*		*	*	*	*	6
Meyerhardt et al (2007)^44^	*		*	*		*	*		*	6
Mehta et al (2017)^45^			*	*	*	*	*	*	*	7
Masaki et al (2003)^46^	*		*	*	*	*	*	*	*	8
Kumagai et al (2014)^47^	*		*	*	*	*	*	*	*	8
Kim et al (2004)^49^	*	*	*	*	*	*	*	*	*	9
Kim et al (2005)^48^	*	*	*	*	*	*	*	*	*	9
Kesse et al (2006)^50^	*		*	*	*	*	*		*	7
Hsiung et al (2016)^51^	*		*	*	*	*	*			6
Fung et al (2014)^52^			*	*	*	*	*		*	6
Fung et al (2012)^56^			*	*	*	*	*	*	*	7
Fung et al (2003)^60^		*	*	*	*	*	*	*	*	8
Flood et al (2008)^59^	*	*	*	*	*	*	*		*	8
Engeset et al (2009)^58^	*	*	*	*		*	*		*	7
Dixon et al (2004)^61^	*	*	*	*	*	*	*	*		8
Butler et al (2008)^62^	*		*	*	*	*	*		*	7
Guo et al (2022)^26^	*		*	*		*	*	*	*	7
Zhao et al (2023)^53^	*		*	*	*	*	*	*	*	8

a An asterisk indicates the study fulfills the respective Newcastle-Ottawa Scale quality appraisal criteria.

### Grading the level of evidence from quantitative analysis

The certainty of evidence for both PCA-derived dietary patterns and the risk of GI cancers was low. However, the certainty of evidence for RRR-derived dietary patterns and GI cancer risk was downgraded to very low due to serious impression, indirectness, and inconsistency (Table S5).

## DISCUSSION

To our knowledge, this systematic review and meta-analysis is the first to compare GI cancer risk and survivorship differences associated with dietary patterns derived by a *posteriori* and *hybrid* methods. Findings included a positive association between PCA-derived unhealthy dietary patterns and a negative association of healthy dietary patterns with GI cancer risk and survivorship. These findings suggest inclusion of fruits, vegetables, whole grains, milk and dairy products, fish, and legumes in the diet may both protect against GI cancer development, and contribute to a reduction in deaths due to GI cancers. Similarly, the estimates also suggest that the RRR-derived healthy dietary pattern is associated with a lower risk of GI cancers, whereas no association was found for the RRR-derived unhealthy dietary pattern. Evidence from PCA-derived dietary patterns and GI cancer risk support current WCRF/AICR recommendations to consume a diet rich in whole grains, vegetables, fruits, and beans and limit consumption of red and processed meat, sugar-sweetened drinks, alcohol, and fast and other processed foods to reduce cancer risk.^8^ Findings from this review suggest it may be important to provide nutrition education in favor of adhering to healthy dietary patterns to reduce the risk of GI cancers and enhance survivorship.

This systematic review and meta-analysis should be considered in light of the following strengths and limitations. First, there is often cross-loading of different food items within each dietary pattern, and the predominant factor loading was used to subjectively define the dietary pattern. As a result, the average weight factor loading was used to objectively identify and classify dietary patterns as healthy or unhealthy. Second, the use of cohort studies provides robust insights into potential causal relationships between dietary intake and the risk of GI cancers when compared with case-control and cross-sectional studies; thus, exclusively focusing on cohort studies in this review is a strength. However, the searches were limited to articles published in English because none of the review team members is proficient in other common languages other than English. Studies may have been published in languages other than English, and excluding them could plausibly have introduced selection bias. Additionally, subgroup analysis based on participant age, cancer stages, or median follow-up time was not possible, due to insufficient data from the studies included. Finally, dose-response analysis was not considered in this review and could be a focus for future studies with more detailed dietary information.

Considering the interpretability of dietary patterns, the degree to which dietary components are loaded, and the degree to which dietary patterns are associated with healthy outcomes,^65^ PCA and RRR seem not to provide the same dietary patterns. This is an important methodological consideration because this highlights that the choice of statistical method used to derive dietary patterns can influence the interpretability of the patterns and the associations observed with health outcomes. This review also demonstrated that PCA-derived dietary patterns were more strongly associated with GI cancer risk than were RRR-derived dietary patterns. In this review, the highest intake of foods that are part of a PCA-derived healthy dietary pattern was associated with a 10% reduced risk of GI cancers compared with the lowest intake. This dietary pattern was characterized by a higher intake of fruits, vegetables, milk and dairy products, whole grains, fish, and coffee. In contrast, the highest intake of PCA-derived unhealthy dietary pattern, characterized by the highest intake of meat and processed meat, refined grains, potato, fast and processed food, sugar-sweetened beverages, salted fish, and alcohol, was associated with a 11% increased risk of GI cancers.

Although only 3 studies were included and results should be interpreted with caution, the estimates also suggest that the RRR-derived healthy dietary pattern was associated with a lower risk of GI cancers, but no association was found for the RRR-derived unhealthy dietary pattern. The use of different types and number of intermediate response variables to derive nutritional data may explain the observed differences here. Willemsen et al^21^ used dietary fiber, vitamin D, fructose, and discretionary fats; Nothlings et al^55^ used quercetin, kaempferol, and myricetin; and Fung et al^60^ used C-peptide as response variables to derive dietary patterns. Even though the selected nutrients are associated with GI cancer risk, they might not fully explain the relationship between diet and cancer compared with specific cancer biomarkers, like C-peptide for CRC.^66^ Furthermore, the use of different cutoff points for factor loadings to quantify the contribution of food groups or items to the dietary patterns may contribute to the observed differences. In this review, the absolute value of weighted average factor loading >0.3 in PCA-derived dietary patterns and without calculating the weighted average factor loading for RRR-derived dietary patterns was used. This may lead to different contributions of food items to dietary patterns and risk estimates between studies. Finally, compared with PCA, RRR uses intermediate response variables that have a specific pathway from diet to disease. Food groups or items that may related to disease risk in different pathways may be excluded.^67^ As a result, this analysis could not draw definitive conclusions about the association between RRR-generated dietary patterns and the risk of GI cancers.

This systematic review and meta-analysis also suggest that consumption of certain foods may improve survival following a diagnosis of GI cancer. A healthy dietary pattern characterized by consuming a diet abundant in fruits, vegetables, whole grains, legumes, and nuts has been associated with improved survival after GI cancer diagnosis.^68^ These nutrient-rich foods are packed with fiber, antioxidants, and phytochemicals, which have demonstrated cancer-protective properties. Additionally, this dietary pattern is recognized for its anti-inflammatory effects and potential to decrease oxidative stress, both of which can contribute to a reduction in cancer mortality.^69^^,^^70^

In contrast, the consumption of a diet high in processed meats, such as sausages, hot dogs, and bacon, as well as red meats like beef and pork, has been associated with an elevated risk of death from GI cancers. These types of meats often contain harmful compounds like nitrates and heme iron, which can contribute to the increased risk of death from GI cancer.^71^ Moreover, diets rich in added sugars, including sugary beverages, desserts, and processed snacks, have been linked to an increased risk of cancer mortality.^72^ Excessive intake of sugar can contribute to weight gain, inflammation, and insulin resistance, all of which can promote the development of other types of cancer and related mortality.^73^

This is the first meta-analysis, to our knowledge, in which average weighted factor loadings guided by the WCRF/AICR are used to compare the GI cancer risk differences associated with dietary patterns derived by a posteriori and hybrid methods, in contrast to previous systematic reviews, which focused on author-reported dietary patterns as presented. However, dietary pattern nomenclature is subjective and depends on the predominant factor loading of food groups/items.^74^ Studies may not have the same factor loading for food items in each dietary pattern, which is prone to classification bias. The reported healthy or unhealthy dietary patterns may not necessarily reflect exactly healthy and unhealthy dietary patterns. As a result, in this review, PCA-derived dietary patterns were classified as healthy and unhealthy based on weighted average factor loadings. The result of this review adds to the knowledge derived from a systematic review conducted previously on dietary patterns and CRC,^75–77^ pancreatic,^78^ gastric^79^ esophageal,^80^ and liver cancer^81^ risks.

In this review, most studies used FFQs, and the length of follow-up ranged from 5 to 32 years to assess the risk of GI cancers, with most of the studies using baseline dietary information to assess cancer risk. The dietary behavior of the study participants might change during the follow-up period. Future studies should consider that the FFQ should include repeated dietary measurements to assess long-term dietary exposure to improve the link between dietary intake and GI cancer risk. Similarly, studies on dietary patterns and GI cancer mortality rely predominantly on single, pre-GI cancer diagnosis records of dietary intake. Change in dietary intake after cancer diagnosis is consequently unclear and unaccounted for; however, among included studies, no cohort study examined whether there was a change in dietary intake and its effect on cancer mortality. Therefore, prospective studies should assess the presence of change in dietary intake and its effect on cancer survival.

The association between dietary patterns and GI cancer risk can be explained by several mechanisms. A healthy dietary pattern that includes fruits, vegetables, whole grains, dairy, and dairy products is a good source of dietary fiber and antioxidants.^82^ Insoluble dietary fiber, in particular, plays a crucial role in promoting the growth of good bacteria in the colon, improving bowel health, and producing short-chain fatty acids that can inhibit tumor formation and abnormal cell growth, stimulate the proliferation of mucosal cells, and improve immune function.^83^ In addition, soluble dietary fiber can congeal to produce a gel that reduces the length of contact of toxic substances with the colon, holds water, and enhances GI motility and gastric emptying.^76^^,^^84^

On the other hand, increased consumption of red and processed meat, pork, potato, processed and fast foods, sugary drinks, and salted food has been linked to excessive body fat accumulation, which may lead to obesity-related cancers.^85^ Additionally, unhealthy dietary patterns may have an impact on the physiological homeostasis of glucose and insulin, which may increase cancer risk.^86^ This is supported by a review of insulin resistance and its contribution to colon carcinogenesis, which showed that insulin resistance is related to cancer risk through increased levels of insulin-like growth factors and hyperinsulinemia.^87^ The unhealthy dietary patterns are characterized by being rich in fat and energy and poor in dietary fiber. Intake of higher amounts of energy-dense fat affects the rate of gastric emptying and colonic transit time and release of hormones or chemicals and distribution of homeostatic regulations.^88^ Higher intake of fat is associated with hepatic synthesis of bile acid, which can increase the risk of GI cancers through oxidative stress and inflammation.^89^ On the other hand, lower amounts of dietary fiber intake may affect the gut microbiota, which can produce an important product that has many advantages, such as anti-inflammatory properties and programmed cell death.^90^

The PCA-derived unhealthy dietary pattern was associated with a higher risk of GI cancers in women. This might be because unhealthy dietary intake may be associated with a higher prevalence of obesity in women compared with men, and its subsequent effect.^91^ Women are less likely to metabolize visceral fats and have a higher level of appetite-regulating hormone leptin than men do.^92^ Higher leptin levels are associated with insulin resistance. Insulin can increase the risk of CRC by its effect on normal and neoplastic colonic cells and the reduced effect of hyperglycemia on the motility of the colon.^93^ The other possible explanation is colonic transit time is faster among men than women,^94^ and this leads to longer exposure to toxic products in women and, finally, chronic disease, including GI cancers.

### Implications and future directions

The first step in determining the association between food intake and health outcomes is collecting accurate dietary data. It is clear that there is still a need for robust, consistent, and clearly reported dietary data between studies, and this has been discussed at length in a previous review.^95^ However, appropriate data analysis is also required to establish a link between dietary intake and health outcomes. This review clarifies that PCA is an important approach to consider when trying to identify food items that explain the most variation in the dietary intake of the study population.^13^ PCA can be used to identify linear composites of food items, food groups, or nutrients that account for the most significant variation in the individual's diet. This approach can generate a relatively small number of components that can account for the variability found in a relatively large number of dietary measures.^12^

The findings from PCA may be important to provide nutrition education to those who are at increased risk of developing GI cancers. Higher consumption of fruits, vegetables, whole grains, legumes, fish, milk, and dairy products, as well as lower intake of red and processed meat, refined grains, salted foods, alcohol, sugar-sweetened beverages, fast and processed food, and potatoes may reduce GI cancer risk.

RRR is also important in explaining the biological mechanisms of the association between dietary patterns and cancer risk.^16^ Compared with PCA, RRR allows for the inclusion of a biomarker as a response variable, which improves the ability to explain how eating habits and chronic diseases are related.^96^^,^^97^ It is used to identify a linear combination of food groups that explain as much variation as possible in a set of intermediate response variables, such as nutrients, metabolic markers, and anthropometric measures.^14^ It is especially important when different types of dietary components are involved in disease onset, progression, and prevention.^98^ Because RRR uses intermediate response variables, it is important to identify dietary patterns related to the disease under investigation, not the dietary behavior of the population. This method is important when there is ethnic-specific dietary intake and specific cancer biomarkers.^99^ Thus, RRR can be used to explore specific biological mechanisms between dietary patterns and GI cancers.^66^ More studies using GI cancer-specific biomarkers as intermediate response variables should be undertaken.

## CONCLUSION

PCA-derived dietary patterns were significantly associated with GI cancer risk and survivorship. PCA-derived healthy dietary patterns characterized by higher intake of fruit, vegetables, whole grains, fish, and milk and dairy products were associated with a reduced risk of GI cancers and better survival, whereas PCA-derived unhealthy dietary patterns characterized by higher intake of red and processed meat, fast and processed food, refined grains, alcohol, and sugar-sweetened beverages had the opposite effect. The RRR-derived healthy dietary pattern may relate to the reduced risk of GI cancers. However, a limited number of studies were included, and the certainty of evidence is very low in this field. Prospective studies considering different nutrients or biomarkers as response variables and GI cancers are needed to further clarify disparities in the association between PCA- and RRR-derived dietary patterns and the risk of GI cancers.

This study adds knowledge to the existing literature by considering factor loading to objectively classify dietary patterns. However, it is important to note that the synthesized evidence in this review ranged from very low to low certainty of evidence. Together, these findings suggest that although an association may be apparent between dietary patterns and GI cancer risk, the quality of evidence to date is low and should be considered with caution.

## Supplementary Material

nuae090_Supplementary_Data
