# Supplementary material for: Difference in Gastrointestinal Cancer Risk and Mortality by Dietary Pattern Analysis: A Systematic Review and Meta-Analysis
Source: Nutr Rev. 2024 Jul 17;83(3):e991–e1013. doi: 10.1093/nutrit/nuae090 (PMC11819480; doi:10.1093/nutrit/nuae090)
Supplement: nuae090_Supplementary_Data [file nuae090_supplementary_data.zip › nuae090_Supplementary_Data/Authors' Response.docx]

**Authors' response**

Manuscript ID= **NUTR-REV-318-SYSR-08-2023.R1**:

**Title: Difference in gastrointestinal cancer risk and mortality by dietary pattern analysis: A systematic review and meta-analysis**

| **Editorial and review comments** | **Response** |
| --- | --- |
| **Editor's comment** |  |
| Changing terms healthy and western, which to contrasting terms. use healthy and unhealthy or traditional and western, or less processed and more processed, or some more parallel set of terms. | Thank you. We used the term “unhealthy” throughout the revised manuscript. |
| **Reviewer #1** |  |
| Please consider refining the language to align with a professional and scientific tone for the intended audience | Thank you. The manuscript has been meticulously edited. |
