# Supplementary material for: Difference in Gastrointestinal Cancer Risk and Mortality by Dietary Pattern Analysis: A Systematic Review and Meta-Analysis
Source: Nutr Rev. 2024 Jul 17;83(3):e991–e1013. doi: 10.1093/nutrit/nuae090 (PMC11819480; doi:10.1093/nutrit/nuae090)
Supplement: nuae090_Supplementary_Data [file nuae090_supplementary_data.zip › nuae090_Supplementary_Data/Supporting Information.docx]

**Title**: Difference in gastrointestinal cancer risk and mortality by dietary pattern analysis: A systematic review and meta-analysis

**First author**: Zegeye Abebe^1, 2^

^1^Flinders University, College of Medicine and Public Health, Flinders Health and Medical Research Institute, Adelaide, South Australia

^2^Department of Human Nutrition, Institute of Public Health, College of Medicine and Health Sciences, University of Gondar, Gondar Ethiopia

**Online Supplemental Information**

**Supplemental Table legends:**

Table S1: Search strategy in Ovid Medline and master search

Table S2: Summary of data extraction from the eligible articles

Table S3: Dietary patterns identified in the original articles and considered for this meta-analysis

Table S4: Reason for exclusion of articles

Table S5: GRADE evidence table for the association between dietary patterns and GI cancer risk and mortality

**Supplemental Figure legends**

Figure S1: Forest plot of subgroup analysis PCA-derived healthy dietary patterns by the sex of the study participants

Figure S2: Forest plot of subgroup analysis PCA-derived healthy dietary patterns and GI cancer types

Figure S3: Forest plot of subgroup analysis PCA-derived Western dietary patterns and GI cancer risk by sex of the study participant

Figure S4: Forest plot of subgroup analysis based on GI cancer types and PCA-derived Western dietary patterns

Figure S5: Forest plot showed the association between PCA-derived healthy dietary pattern and colon and rectum cancer

Figure S6: Forest plot showed the association between PCA-derived western dietary pattern and colon and rectum cancer

Figure S7: Funnel plot to assess the presence of publication bias among the studies included in PCA-derived healthy dietary pattern and GI cancer risk

Figure S8: Funnel plot to assess Publication bias among those studies included in western dietary patterns and GI cancer risk

Figure S9: Funnel plot to assess Publication bias among those studies included in RRR-derived healthy dietary patterns and GI cancer risk

Figure S10: Funnel plot to assess Publication bias among those studies included in RRR-derived western dietary patterns and GI cancer risk

Table S1: Search strategy in Ovid Medline and master search

| # | Medline | Master search |
| --- | --- | --- |
| 1 | exp neoplasms/ | exp neoplasms/ |
| 2 | ((Cancer or Neoplasms or Digestive system or Gastrointestinal or Esophageal or Esophageal or Squamous Cell or Intestin* or Cecal or Appendiceal or Appendix or Colorectal or Colon* or Colitis-Associated or Sigmoid or Rectal or Rectum or Anal or Anus or Duodenal or Ileal or Gastric or Hepto* or Heptic* or Hepta* or Liver or Pancreatic or Islet Cell or Stomach or bowel) adj3 (cancer* or neoplas* or tumour or tumor or carcinoma or metast* or Adenoma or adenocarcinoma)).tw,kf. | ((Cancer OR Neoplasms OR "Digestive system" OR Gastrointestinal OR Esophageal OR Esophageal OR "Squamous Cell" OR Intestin* OR Cecal OR Appendiceal OR Appendix OR Colorectal OR Colon* OR "Colitis-Associated" OR Sigmoid OR Rectal OR Rectum OR Anal OR Anus OR Duodenal OR Ileal OR Gastric OR Hepto* OR Heptic* OR Hepta* OR Liver OR Pancreatic OR Islet Cell OR Stomach OR bowel) adj3 (cancer* OR neoplas* OR tumour OR tumor OR carcinoma OR metast* OR Adenoma OR adenocarcinoma)) |
| 3 | (Adenomatous Polyposis Coli or Gardner Syndrome).tw,kf. | (Adenomatous Polyposis Coli OR Gardner Syndrome) |
| 4 | or/1-3 | 1 OR 2 OR 3 |
| 5 | cluster analysis/ or factor analysis, statistical/ or principal component analysis/ | cluster analysis/ OR factor analysis, statistical/ OR principal component analysis/ |
| 6 | data mining/ or multifactor dimensionality reduction/ | data mining/ OR multifactor dimensionality reduction/ |
| 7 | (Posteriori methods or Hybrid methods or Principal component analysis or Rank reduced regression or reduced rank regression or stepwise linear regression or Cluster analysis or Data driven or Factor analysis or Partial least square or Data mining or Treelet transformation or Least absolute shrinkage or selection operator or LASSO).tw,kf. | ("Posteriori methods" OR "Hybrid methods" OR "Principal component analysis" OR "Rank reduced regression" OR "reduced rank regression" OR "stepwise linear regression" OR "Cluster analysis" OR "Data driven" OR "Factor analysis" OR "Partial least square" OR "Data mining" OR "Treelet transformation" OR "Least absolute shrinkage and selection operator" OR LASSO) |
| 8 | or/5-7 | 5 OR 6 OR 7 |
| 9 | Diet, Healthy/ | Diet, Healthy/ |
| 10 | Diet, Western/ | Diet, Western/ |
| 11 | Feeding Behavior/ | Feeding Behavior/ |
| 12 | ((eat* or diet or feeding) adj3 (western or unhealth* or health* or prudent or pattern* or index* or quality or habbit* or pattern or behaviour* or behavior*)).tw,kf. | ((eat* OR diet OR feeding) adj3 (western OR unhealth* OR health* OR prudent OR pattern* OR index* OR quality OR habbit* OR pattern OR behaviour* OR behavior*)) |
| 13 | or/9-12 |  |
| 14 | 4 and 8 and 13 | 4 AND 8 AND 13 |

**Table S2**: Summary of data extraction from the eligible articles

| **Domains** | **Data to be extracted** |
| --- | --- |
| General | Name of the first author  Year of publication  type of gastrointestinal cancer |
| Methods | Study area/country  Study design  Study participants  Methods of dietary pattern analysis  The measure of the association between dietary patterns and cancer risks  Tools used for measuring dietary exposure |
| Results | Cases and the total number of participants  Age group of study participants when applicable  Sex  Estimates of association with 95%CI  Dietary patterns and food components  Factor loading |

**Table S3:** Dietary patterns identified in the original articles and considered for this meta-analysis

| References | Methods of Dietary pattern analysis | Dietary patterns in the original paper | Food components | Dietary pattern considered for this meta-analysis |
| --- | --- | --- | --- | --- |
| Zhang 2013 | PCA | Vegetable-based dietary pattern | Characterized by a high intake of vegetables | Healthy |
|  |  | Fruit-based dietary pattern | Characterized by a high intake of fresh fruits | Healthy |
|  |  | Meat-based dietary pattern | Characterized by a high intake of meat, poultry, and animal parts (heart, brain, tongue, intestine | Western |
| Wirfalt 2009 | CA | Many foods | For men and women, did not indicate any specific distinguishing food, but intakes of alcohol and sweets ranked comparatively high | Excluded from this meta-analysis |
|  |  | Vegetables and fruits | For men and women, high intakes of vegetables, fruits, and low-fat foods like fish and lean chicken. | Excluded from this meta-analysis |
|  |  | Fatty meats | (For men only) Characterized by regular-fat meats | Excluded from this meta-analysis |
|  |  | Fat-reduced foods | fat-reduced foods (but not lean meats), with skim milk ranking comparatively high (for men only) | Excluded from this meta-analysis |
|  |  | Diet foods, lean meats | characterized by diet foods and lean meats (FOR FEMALE ONLY) | Excluded from this meta-analysis |
| Willemsen 2022 | PCA | Western | characterized by a high intake of grain servings, especially non-whole grains, vegetables, white potatoes, cheese, meats from lamb, pork, beef, and luncheon meats (red and processed meats), discretionary fats, and teaspoons of added sugar. | Western |
|  |  | Prudent | characterized by a high intake of vegetables, fruits, and lean meat from fish and other seafood | Healthy |
|  |  | sugar, fruits, and dairy | comprised of grain servings, especially whole grains, fruits, dairy, and teaspoons of added sugar | Healthy |
| Willemsen 2022 | RRR | Dietary fiber | Intake of grain servings, vegetables, and fruits | Healthy |
|  |  | Vitamin D | dairy and fish and other seafood | Healthy |
|  |  | Fructose | Fruits and teaspoons of added sugar | Western |
|  |  | Discretionary fats | Intake of excess solid fats present within the “Milk” and “Meat and Beans” categories (e.g., whole vs. skim milk) | Western |
| Wie 2017 | PCA | Vegetables and fish | characterized by a high intake of oils, vegetables, fish and shellfish, eggs, seaweed, and mushrooms | Healthy |
|  |  | Rice and kimchi | Loaded positively for rice, soybean paste, and kimchi, but negatively for noodles, dumplings, bread, and snacks | Healthy |
|  |  | Fruits and dairy | High positive loading for fruits, milk, dairy products, other grains, potatoes, and nuts, | Healthy |
|  |  | Meats and sweets | characterized by high consumption of alcohol, red and white meat and animal products, and sweets | Western |
| VanBlarigan 2020 | PCA | Prudent | high intake of vegetables, legumes, and fruit | Health |
|  |  | Western | higher intake of dairy, refined grains, condiments, red meat, and sweets and desserts | Western |
| Thordardottir 2022 | PCA | Traditional | characterized by high consumption of salted or smoked meat, salted or smoked fish, blood and liver sausage (offal), rye bread, milk and milk products, and oatmeal | Western |
|  |  | pattern 2 | characterized by high consumption of fruit, vegetables, and fish as topping on bread and in salad | Healthy |
|  |  | pattern 3 | had a positive factor loading for fish oil, oatmeal, and blood and liver sausage (offal | Healthy |
|  |  | pattern 4 | had positive factor loadings for meat, fish, and potatoes | Western |
| Terry 2001 | PCA | Healthy | fruits and vegetables, fish and poultry, cereal and whole-grain bread, fruit juice, and low-fat dairy products | Healthy |
|  |  | Western | intakes of foods associated with a Western diet: processed and red meats, soda and sweets, refined bread and potatoes, and high-fat dairy products | Western |
|  |  | Drinker | primarily reflected the correlated intakes of wine, beer, and spirit | Western |
| Shin 2018 | PCA | Prudent | characterized by high intakes of vegetables, fruit, noodle, potatoes, soy products, mushrooms, and seaweed | Healthy |
|  |  | Western | heavily loaded with meat and processed meat, eel, dairy foods, fruit juice, coffee, tea, soft beverages, sauces, and alcohol | Healthy |
|  |  | Traditional | high loadings of pickles, seafood, fish (oily-, salty-, lean fish, and salmon), chicken, and sake (males only) | Healthy (females only) |
| Sharma 2018 | PCA | processed Meat | Red meat, Cured/processed red meat, fish, Processed Fish | Western |
|  |  | Prudent Vegetable | Cruciferous vegetables, other fruit, other greens, other vegetables, whole grains, dried fruit, cereals, | Healthy |
|  |  | High sugar | Desserts and Sweets, Pies, Tarts | Will not be used |
| Nothlings 2008 | RRR | quercetin, kaempferol, and myricetin | tea, cabbages, fresh fruit, wine, quercetin, kaempferol, myricetin | Healthy |
| Michaud 2005 | PCA | Prudent | vegetables, legumes, fruit, whole grains, fish, and poultry | Healthy |
|  |  | Western | Characterized by high consumption of red meat, processed meat, refined grains, French fries, high-fat dairy products, sweets and desserts, and high-sugar drinks | Western |
| Meyerhardt 2007 | PCA | Prudent | Characterized by high intakes of fruits, vegetables, whole grains, legumes, poultry, and fish | Healthy |
|  |  | Western | Characterized by refined grains, processed and red meats, desserts, high-fat dairy products, and french fries | Western |
| Mehta 2017 | PCA | Prudent | Characterized by a high intake of vegetables, fruits, whole grains, and legumes | Healthy |
|  |  | Western | Characterized by red and processed meats, refined grains, and desserts | Western |
| Masaki 2003 | PCA | vegetable and fruit | Loaded greatly on cabbage, lettuce, green leafy vegetables, carrots, oranges and other fruits. | Healthy |
|  |  | Western breakfast | Bread, butter, cheese, hum sausage, and coffee were consumed more often in contrast to rice, seaweeds, bean curd, and pickled vegetables | Excluded from this meta-analysis |
|  |  | Meat | Positively loaded on pork, beef, and chicken and negatively on tomatoes and other fruits | Western |
|  |  | Rice/snack | rice, miso soup, cookies, and orange juice loaded heavily in contrast to negative levels of vegetables | healthy |
| Kumagai 2014 | PCA | Japanese dietary pattern | Egg, deep-fried dishes, tempura, fried vegetables, raw fish, fish boiled with soy, roast fish, boiled fish paste, dried fish, green vegetables, carrots, pumpkin, tomato, cabbage, lettuce, mushroom, soybean, orange, | Healthy |
|  |  | Animal food | beef, pork, ham, chicken, liver | Western |
|  |  | high-dairy, high-fruit-and vegetable, and low-alcohol (DFA) | MILK, Rice, miso soup, yogurt, margarine, alcohol | Healthy |
| Kim 2004 | PCA | Healthy | heavily loaded with vegetables, fruits, soy products, seaweeds, mushrooms, milk, beans, and yogurt | Healthy |
|  |  | Traditional | loaded with pickled vegetables, salted fish and roe, fish, rice, and miso soup for both genders with a negative loading for bread and butter. additionally loaded with alcoholic beverages (sake, shochu and beer) for men | Healthy (women only) |
|  |  | Western | loaded with meat, poultry, cheese, bread, and butter | Western |
| Kim 2005 | PCA | Healthy | heavily loaded with vegetables, fruits, soy products, seaweeds, mushrooms, milk, beans, and yogurt | Healthy |
|  |  | Traditional | loaded with alcoholic beverages (sake, shochu, and beer) | Healthy (women only) |
|  |  | Western | loaded with meat, poultry, cheese, bread, butter | Western |
| Kesse 2006 | PCA | Healthy | characterized by high consumption of raw and cooked vegetables, legumes, fruit, yogurt, fresh cheese, breakfast cereals, sea products, eggs, and vegetable oils (olive oil and others) and by low consumption of sweets | Healthy |
|  |  | Western | positively correlated with the consumption of potatoes, pizza and pie, sandwiches, legumes, sweets, cakes, cheese, bread, rice, pasta, processed meat, eggs, and butter | Western |
|  |  | Drinker | associated with a high consumption of sandwiches, snacks, coffee, processed meat, sea products, wine, and other alcoholic beverages, as well as a low consumption of soup and fruit | Western |
|  |  | Meat eaters | positively associated with high consumption of potatoes, legumes, coffee, meat, poultry, vegetable oils (except olive oil), and margarine and negatively associated with tea, olive oil, and breakfast cereals | Western |
| Hsiung 2016 | PCA | salted foods | Fresh salted fish intake (most exposed), Fresh seashell intake (most exposed), Cooked seashell intake (most exposed), Fresh seafood intake (most exposed), Cooked seafood intake (most exposed), Salted meat intake, Salted vegetable intake (most exposed | western |
|  |  | Seafood | Seafood intake (most exposed), Cooked salted fish intake (most exposed), Shrimp sauce intake | healthy |
|  |  | vegetables and beans | Fermented bean intake (most exposed), Peanut intake (most exposed), Leaf vegetable intake (most exposed), Root vegetable intake (most exposed), Bean intake (most exposed) | Healthy |
|  |  | Meat | Meat | Western |
|  |  | fish foods | Fish noodle intake (most exposed), Fish ball intake (most exposed), fish intake (most exposed) | Healthy |
|  |  | Milk | Milk intake (age<15), Milk intake (age 16-30), Milk intake (age >30) | Healthy |
|  |  | Fruit and leaf vegetables | Leaf vegetable intake (most exposed), Fruit intake | Healthy |
|  |  | Shrimp sauce | Shrimp sauce intake (age 16-30), shrimp sauce intake (age>30) | Will not be used |
| Fung 2014 | PCA | Prudent | Characterized by higher intakes of fruits, vegetables, whole grains, poultry, and low-fat dairy products | Healthy |
|  |  | Western | characterized by higher intakes of red and processed meats, refined grains, sweets and desserts, and high-fat dairy products | Western |
| Fung 2012 | RRR | C-peptide dietary pattern score | characterized by higher meat, fish, and sweetened beverage intake, but lower coffee, high-fat dairy, and whole grains intake | Western |
| Fung 2003 | PCA | Prudent | characterized by high intakes of fruits, vegetables, whole grains, legumes, poultry, and fish | Healthy |
|  |  | Western | characterized by refined grains, processed and red meats, desserts, high-fat dairy products, and French fries | Western |
| Flood 2008 | PCA | Fruit and vegetables | broccoli, lettuce, carrots, tomatoes, pepper, tomatoes, orange, apple | Healthy |
|  |  | Fat-reduced/diet foods | milk, margarine, crackers, cold cut, chicken, mayonnaise, Turkey | Healthy (women only) |
|  |  | Meat and potatoes | meatloaf, beef stew, pork, gravy, bacon | Western |
| Engeset 2009 | CA | Fish | Fish on bread, Sour cream, full fat on fish, sour cream, reduced fat on fish, sauce without fat on fish, sauce with fat on fish, fish liver, fish roe, fish products, fatty fish, lean, white fish, potatoes, carrot, boiled coffee | Excluded from this meta-analysis |
|  |  | Healthy | Skimmed milk, yoghurt, juice, cereals, rice, chicken, fruit, and cod liver oil | Excluded from this meta-analysis |
|  |  | Average | Breakfast cereals, white bread, jam, fruit, potatoes, desserts, Bakery products, | Excluded from this meta-analysis |
|  |  | Western | Higher intake of meat products, bakery products, desserts, and chocolate, and more modern products like pizza, rice, and pasta | Excluded from this meta-analysis |
|  |  | Bread | Typical breakfast and lunch habits in Norway with milk, course bread, jam, cheese and fat on bread | Excluded from this meta-analysis |
|  |  | Alcohol | Beer, wine, and liquor | Excluded from this meta-analysis |
| Dixon 2004 | PCA | Vegetables | Characterized by intakes of vegetables and legumes, citrus fruit and berries, pasta and rice, poultry and fish, and oil and salad dressings and was correlated with intakes of vitamins A, C, and E; folate; and polyunsaturated fatty acids | Healthy |
|  |  | Pork, Processed Meats, Potatoes (PPP) | Characterized by intakes of pork, processed meats, potatoes, and coffee, and was correlated with intakes of energy, protein, carbohydrate, fat, saturated and monounsaturated fatty acids, cholesterol, B vitamins, and minerals | Western |
| Butler 2008 | PCA | Meat- dim sum | characterized by vegetable, fruit, and soy food intake; of the 32 foods included in the pattern, 23 were vegetables, five were soy food items, and four were fruit items. | Western |
|  |  | Vegetable- fruit- soy | The meat-dim sum pattern contained 31 food items, predominantly chicken, pork, fish, rice, and noodle dishes, and preserved foods. | Healthy |
| Guo 2022 | PCA | Prudent | high consumption of salad raw vegetables, cooked vegetables, fresh fruit, dried fruit, oily fish, and non-oily fish | Healthy |
|  |  | Western | high consumption of processed meat, poultry, beef, lamb mutton, and pork | Western |
| Zhao 2022 |  | Pattern 1 | characterized with a higher loading of fresh vegetables, fruits, bean and products, and nuts | Healthy |
|  |  | Pattern 2 | characterized with a higher loading of preserved vegetables, pickled vegetables and salted meat | Western |
|  |  | Pattern 3 | white meat and fish and shrimp, | Healthy |

**Table S4**: Reason for exclusion for articles

| S.NO | Authos | Title | Reason for exclusion |
| --- | --- | --- | --- |
|  | Abdelrehim 2018 | Dietary Factors Associated with Pancreatic Cancer Risk in Minia, Egypt: Principal Component Analysis | Case-control study |
|  | Amtha 2009 | Dietary patterns and risk of oral cancer: a factor analysis study of a population in Jakarta, Indonesia | Case-control study |
|  | Austin 2007 | A diet high in fruits and low in meats reduces the risk of colorectal adenomas | Case-control study |
|  | Bahmanyar 2006 | Dietary patterns and risk of squamous-cell carcinoma and adenocarcinoma of the esophagus and adenocarcinoma of the gastric cardia: a population-based case-control study in Sweden | Case-control study |
|  | Bertuccio 2009 | Nutrient dietary patterns and gastric cancer risk in Italy | Case-control study |
|  | Bosetti 2013 | Nutrient-based dietary patterns and pancreatic cancer risk | Case-control study |
|  | Botma 2013 | Dietary patterns and colorectal adenomas in Lynch syndrome: The GEOLynch cohort study | Wrong outcome (Adenoma) |
|  | Bravi 2010 | Nutrient dietary patterns and the risk of colorectal cancer: a case-control study from Italy | Case-control study |
|  | Bravi 2012 | Dietary patterns and the risk of esophageal cancer | Case-control study |
|  | Campbell 2008 | Dietary patterns and risk of incident gastric adenocarcinoma | Case-control study |
|  | Castello 2019 | Low adherence to the western and high adherence to the mediterranean dietary patterns could prevent colorectal cancer | Case-control study |
|  | Castello 2018 | High adherence to the Western, Prudent, and Mediterranean dietary patterns and risk of gastric adenocarcinoma: MCC-Spain study | Case-control study |
|  | Chan 2013 | Dietary patterns and risk of pancreatic cancer in a large population-based case-control study in the San Francisco Bay Area | Case-control study |
|  | Chen 2002 | Dietary patterns and adenocarcinoma of the esophagus and distal stomach | Case-control study |
|  | Chen 2015 | Dietary patterns and colorectal cancer: results from a Canadian population-based study | Case-control study |
|  | Cho 2018 | Inflammatory Dietary Pattern, IL-17F Genetic Variant, and the Risk of Colorectal Cancer | Case-control study |
|  | Cottet 2005 | Dietary patterns and the risk of colorectal adenoma recurrence in a European intervention trial | Wrong outcome (Adenoma) |
|  | Dalmartello 2020 | Dietary patterns and oral and pharyngeal cancer using latent class analysis | Case-control study |
|  | Dalmartello 2021 | Dietary patterns and oesophageal cancer: a multi-country latent class analysis | Case-control study |
|  | DeStefani 2013 | Dietary patterns and risk of cancers of the upper aerodigestive tract: a factor analysis in Uruguay | Case-control study |
|  | DeStefani 2008 | Nutrient patterns and risk of squamous cell carcinoma of the esophagus: a factor analysis in uruguay | Case-control study |
|  | DeStefani 2005 | Dietary patterns and risk of cancer of the oral cavity and pharynx in Uruguay | Case-control study |
|  | DeStefani 2008 | Exploratory factor analysis of squamous cell carcinoma of the esophagus in Uruguay | Case-control study |
|  | DeStefani 2004 | Dietary patterns and risk of gastric cancer: a case-control study in Uruguay | Case-control study |
|  | DeStefani 2009 | Dietary patterns and risk of cancer: a factor analysis in Uruguay | Case-control study |
|  | deToledo 2010 | Dietary patterns and risk of oral and pharyngeal cancer: a case-control study in Rio de Janeiro, Brazil | Case-control study |
|  | DeVito 2019 | Shared and Study-specific Dietary Patterns and Head and Neck Cancer Risk in an International Consortium | Case-control study |
|  | Denova-GutiÃ©rrez 2014 | Dietary Patterns and Gastric Cancer Risk in Mexico | Case-control study |
|  | Edefonti 2010 | Nutrient-based dietary patterns and the risk of oral and pharyngeal cancer | Case-control study |
|  | Edefonti 2012 | Nutrient-based dietary patterns and the risk of head and neck cancer: a pooled analysis in the International Head and Neck Cancer Epidemiology consortium | Case-control study |
|  | Entwistle 2021 | Dietary patterns related to total mortality and cancer mortality in the United States | wrong outcome (all cancer mortality) |
|  | Hajizadeh 2012 | Nutrient patterns and risk of esophageal squamous cell carcinoma: a case-control study | Case-control study |
|  | Hajizadeh 2010 | Dietary patterns and risk of oesophageal squamous cell carcinoma: a case-control study | Case-control study |
|  | Haslam 2018 | Association between dietary pattern scores and the prevalence of colorectal adenoma considering population subgroups | Cross-sectional study; |
|  | Heidemann 2008 | Dietary patterns and risk of mortality from cardiovascular disease, cancer, and all causes in a prospective cohort of women | Wrong outcome (all cancer mortality) |
|  | Helen-Ng 2012 | Dietary pattern and oral cancer risk--a factor analysis study | Case-control study |
|  | Hu 2022 | Dietary fatty acid patterns and risk of oesophageal squamous cell carcinoma | Case-control study |
|  | Ibiebele 2012 | Dietary patterns and risk of oesophageal cancers: a population-based case-control study | Case-control study |
|  | Katsidzira 2018 | Dietary patterns and colorectal cancer risk in Zimbabwe: A population-based case-control study | Case-control study |
|  | Kim 2021 | Dietary patterns and gastric cancer risk in a Korean population: a case-control study | Case-control study |
|  | Kurotani 2010 | Dietary patterns and colorectal cancer in a Japanese population: the Fukuoka Colorectal Cancer Study | Case-control study |
|  | Lan 2018 | Dietary patterns and primary liver cancer in chinese adults: A case-control study | Case-control study |
|  | Lin 2014 | A dietary pattern rich in lignans, quercetin and resveratrol decreases the risk of oesophageal cancer | Case-control study |
|  | Liu 2017 | Dietary patterns and the risk of esophageal squamous cell carcinoma: A population-based case-control study in a rural population | Case-control study |
|  | MagalhÃ£es 2011 | Dietary patterns and colorectal cancer: A case-control study from Portugal | Case-control study |
|  | Marchioni 2007 | Dietary patterns and risk of oral cancer: A case-control study in SÃ£o Paulo, Brazil | Case-control study |
|  | Miller 2010 | Dietary patterns, red and processed meat-derived mutagen exposure, and colorectal cancer risk | Case-control study |
|  | Miller 2010 | Diet index-based and empirically derived dietary patterns are associated with colorectal cancer risk | Case-control study |
|  | Mizoue 2005 | Dietary patterns and colorectal adenomas in Japanese men: the Self-Defense Forces Health Study | Cross-sectional study |
|  | Moussa 2021 | Dietary Patterns and Hepatocellular Carcinoma Risk among US Adults | Case-control study |
|  | Narmcheshm 2022 | Patterns of Nutrient Intake in Relation to Gastric Cancer: A Case Control Study | Case-control study |
|  | NavarroSilvera 2004 | Dietary and Lifestyle Patterns and Risk of Subtypes of Esophageal and Gastric Cancer: Factor Analysis | Case-control study |
|  | NavarroSilvera 2011 | Principal component analysis of dietary and lifestyle patterns in relation to risk of subtypes of esophageal and gastric cancer | Case-control study |
|  | Nimptsch 2014 | Dietary patterns during high school and risk of colorectal adenoma in a cohort of middle-aged women | Wrong outcome (Adenoma) |
|  | Nkondjock 2005 | Dietary patterns and risk of pancreatic cancer | Case-control study |
|  | Palli 2001 | Dietary patterns, nutrient intake and gastric cancer in a high-risk area of Italy | Case-control study |
|  | Park 2016 | Dietary patterns and colorectal cancer risk in a Korean population: A case-control study | Case-control study |
|  | Park 2016 | Dietary patterns and colorectal cancer risk in a Korean population | Case-control study |
|  | Pou 2012 | Applying multilevel model to the relationship of dietary patterns and colorectal cancer: an ongoing case-control study in CÃ³rdoba, Argentina | Case-control study |
|  | Pou 2014 | [Cancer and its association with dietary patterns in Cordoba (Argentina)] | Non-English language and case-control study |
|  | Randall 1992 | Dietary Patterns and Colon Cancer in Western New York | Case-control study |
|  | Rouillier 2005 | Dietary patterns and the adenomacarcinoma sequence of colorectal cancer | Case-control study |
|  | Safari 2013 | Dietary patterns and risk of colorectal cancer in Tehran Province: a case-control study | Case-control study |
|  | Sewram 2014 | Diet and esophageal cancer risk in the Eastern Cape Province of South Africa | Case-control study |
|  | Shahril 2021 | 'Energy-Dense, High-SFA and Low-Fiber' Dietary Pattern Lowered Adiponectin but Not Leptin Concentration of Breast Cancer Survivors | Cross-sectional study |
|  | Slattery 1998 | Eating patterns and risk of colon cancer | Case-control study |
|  | Stefani 2011 | Dietary patterns and risk of colorectal cancer: a factor analysis in uruguay | Case-control study |
|  | Tayyem 2017 | Dietary patterns and colorectal cancer | Case-control study |
|  | Tseng 1999 | "Eating patterns and risk of colon cancer" | Letters to the Editor |
|  | Wei 2021 | Association of Meal and Snack Patterns With Mortality of All-Cause, Cardiovascular Disease, and Cancer: The US National Health and Nutrition Examination Survey, 2003 to 2014 | Wrong outcome (all cancer mortality) |
|  | Woo 2013 | Glycemic index and glycemic load dietary patterns and the associated risk of breast cancer: a case-control study | Case-control study |
|  | Zang 2022 | Dietary patterns and severity of symptom with the risk of esophageal squamous cell carcinoma and its histological precursor lesions in China: a multicenter cross-sectional latent class analysis | Cross-sectional study; |
|  | Zhuo 1999 | Factor analysis of digestive cancer mortality and food consumption in 65 Chinese counties | Case-control study |
|  | Ziervogel 2010 | Dietary patterns and risk of pancreatic cancer in a hospital-based case-control study | Case-control study |
|  | Gunathilake 2022 | Association between dietary intake networks identified through a Gaussian graphical model and the risk of cancer: a prospective cohort study | All cancer risk |
|  | Okada 2016 | Dietary Patterns and Risk of Esophageal Cancer Mortality: The Japan Collaborative Cohort Study | Cancer mortality not cancer survival |
|  | Pham 2010 | Dietary patterns and risk of stomach cancer mortality: the Japan collaborative cohort study | Cancer mortality not cancer survival |

**Table S5**: GRADE evidence table for the association between dietary patterns and GI cancer risk and mortality

| Certainty assessment: GI cancer risk | | | | | | | Other criteria considered | Effect | Certainty |
| --- | --- | --- | --- | --- | --- | --- | --- | --- | --- |
| No of studies | Design | Risk of bias | Inconsistency/  heterogeneity | Indirectness | Imprecision | Publication bias | Dose-response analysis | Relative Risk (RR)  (95% CI) |  |
| PCA-derived healthy dietary patterns | | | | | |  |  |  |  |
| 21 | Cohort | low | Not Serious | Not Serious | Not Serious | None | None | **0.93** (0.87, 0.98) | low |
| PCA-derived western dietary patterns | | | | | |  |  |  |  |
| 21 | Cohort | low | Not Serious | Not Serious | Not Serious | None | None | **1.14** (1.07, 1.22) | low |
| RRR-derived healthy dietary patterns | | | | | |  |  |  |  |
| 3 | Cohort | low | Serious ^a^ | Serious ^b^ | Serious ^c^ | None | None | **0.83**  (0.61, 1.12) | Very low |
| RRR-derived western dietary patterns | | | | | |  |  |  |  |
| 3 | Cohort | low | Serious ^a^ | Serious ^b^ | Serious ^c^ | None | None | 0.93  (0.57 to 1.52) | Very low |

Explanations

a. Serious inconsistency since moderate heterogeneity: Downgraded


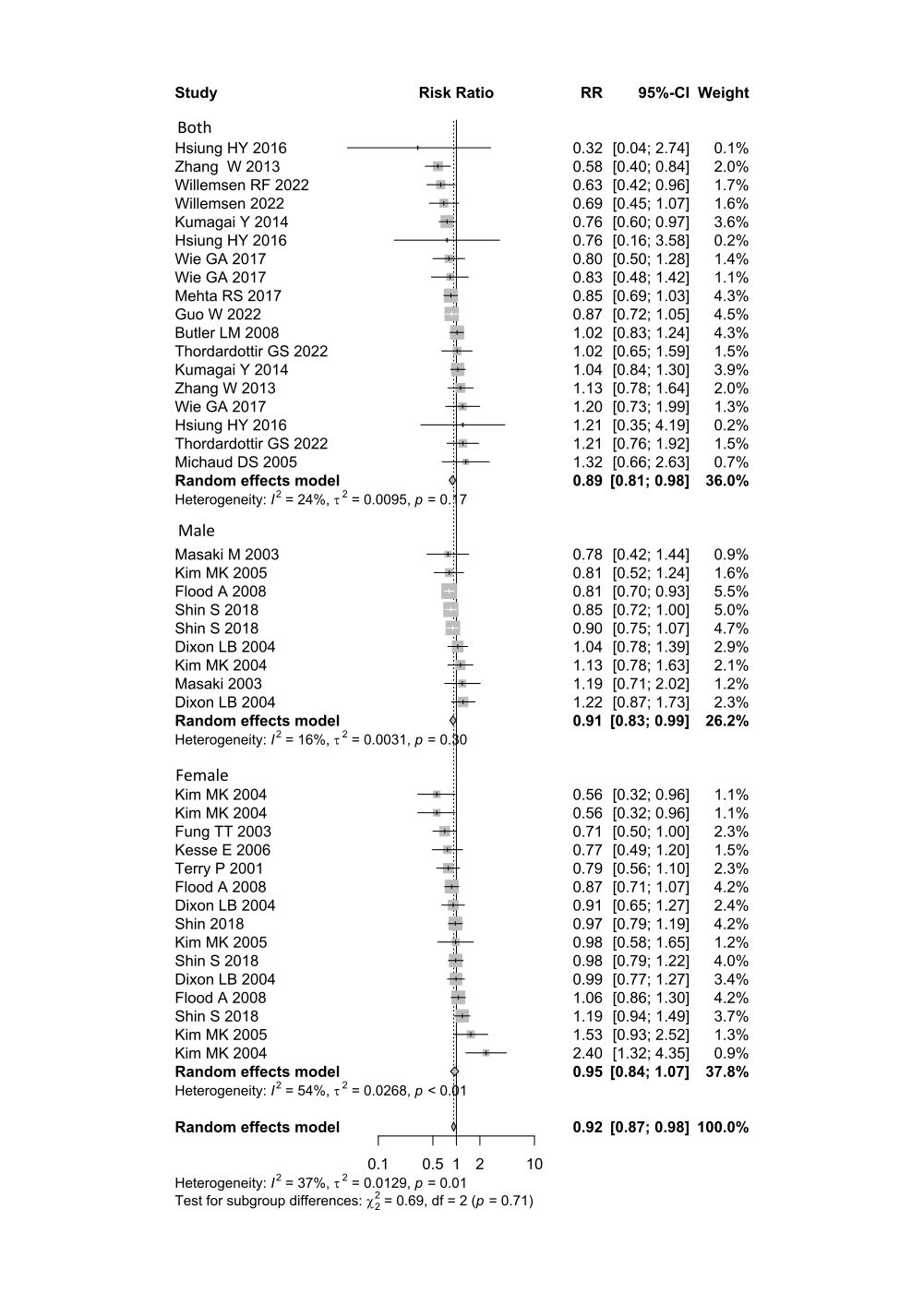


**Figure S1**: Forest plot of subgroup analysis PCA-derived healthy dietary patterns by the sex of the study participants


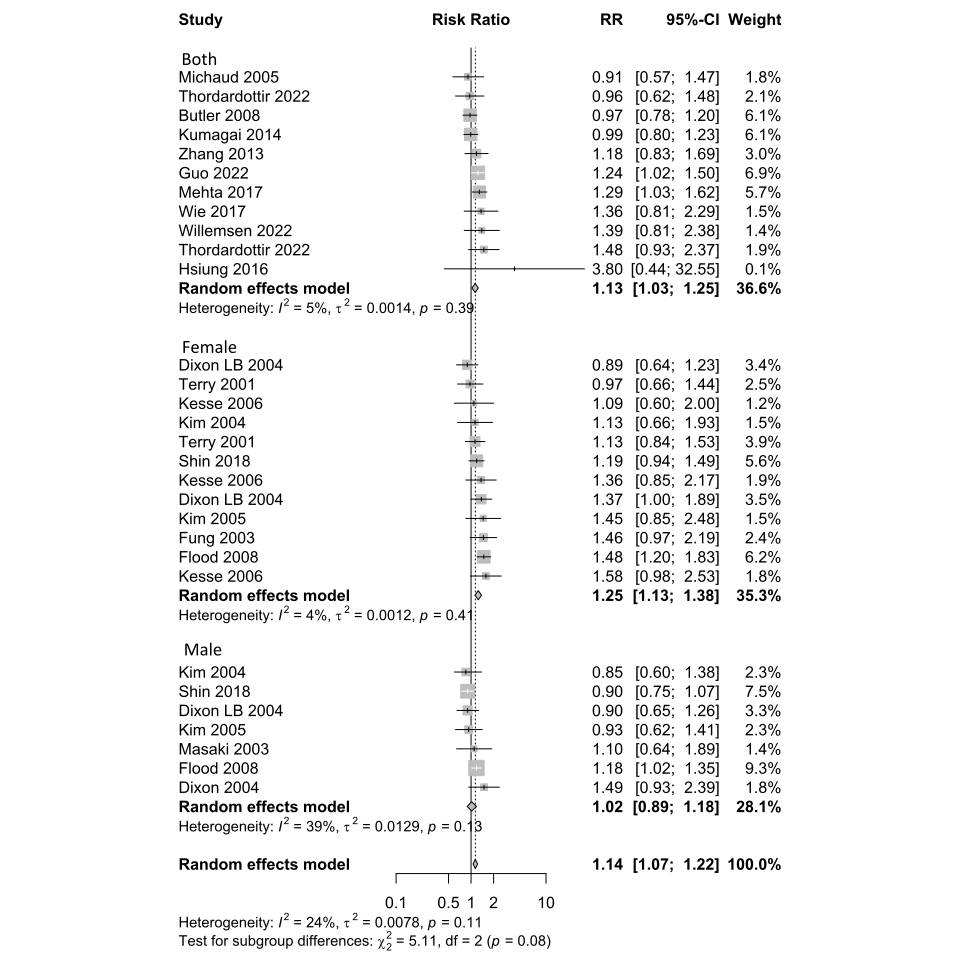


**Figure** S2: Forest plot of subgroup analysis PCA-derived Western dietary patterns and GI cancer risk by sex of the study participant


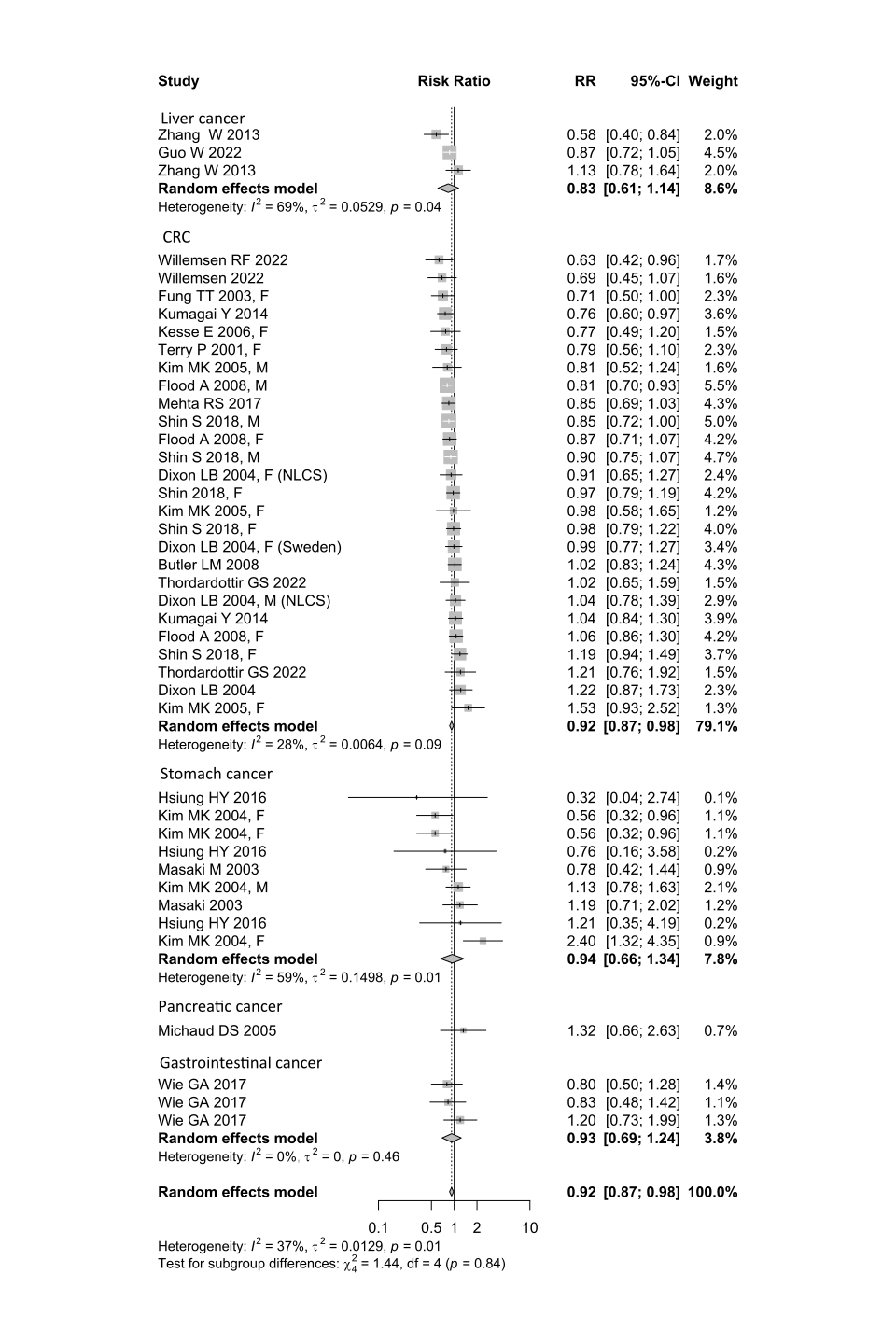


**Figure** S3: Forest plot of subgroup analysis PCA-derived healthy dietary patterns and GI cancer types


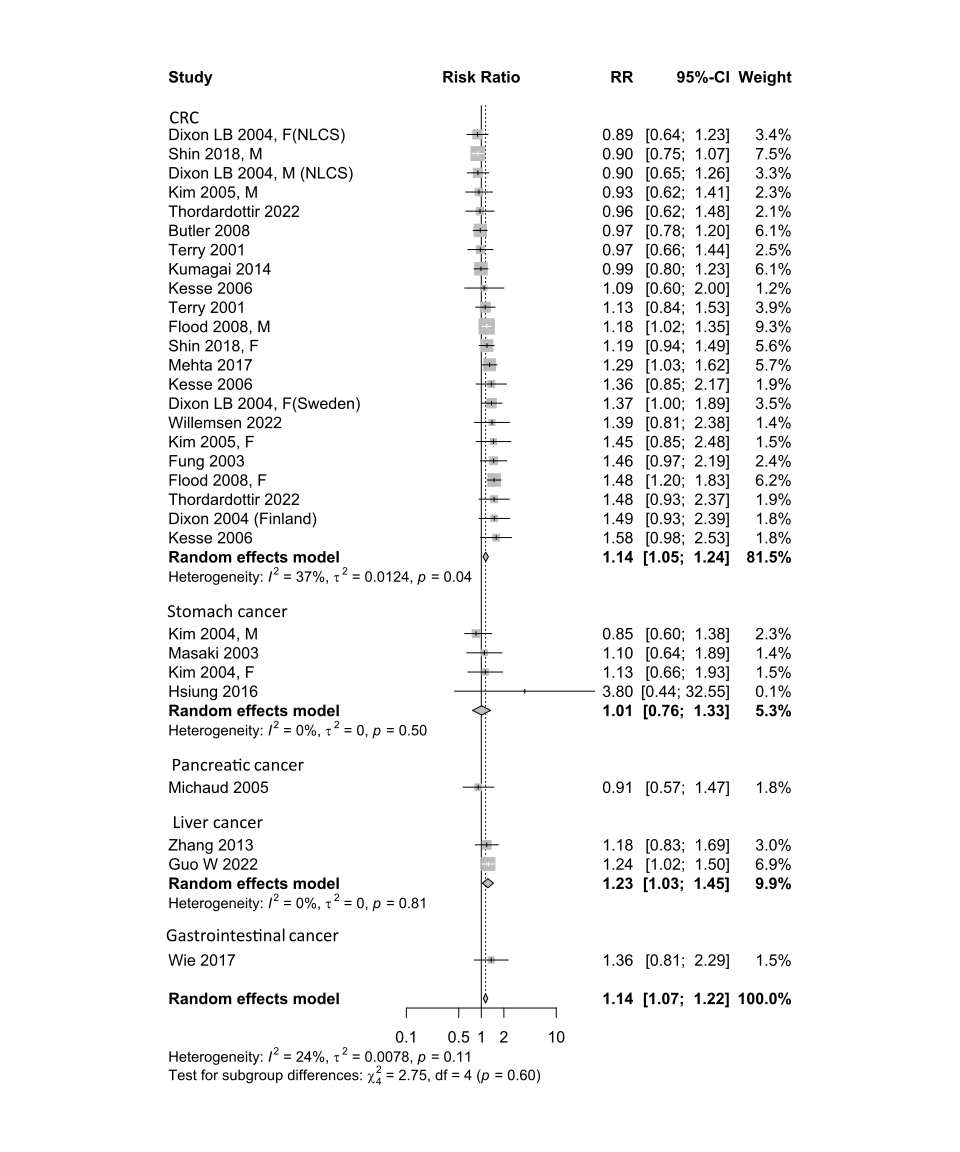


**Figure** S4: Forest plot of subgroup analysis based on GI cancer types and PCA-derived Western dietary patterns


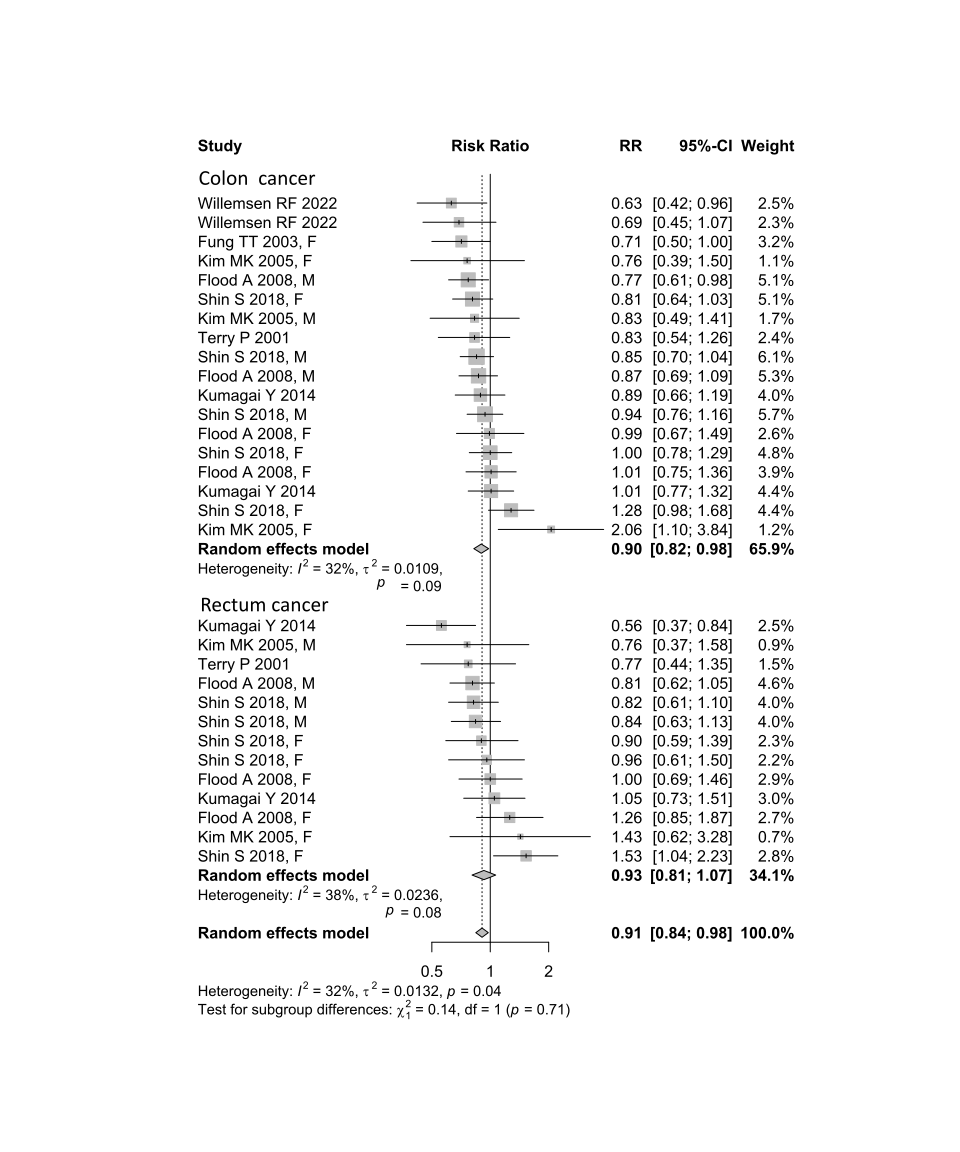


**Figure** S5: Forest plot showed the association between PCA-derived healthy dietary pattern and colon and rectum cancer


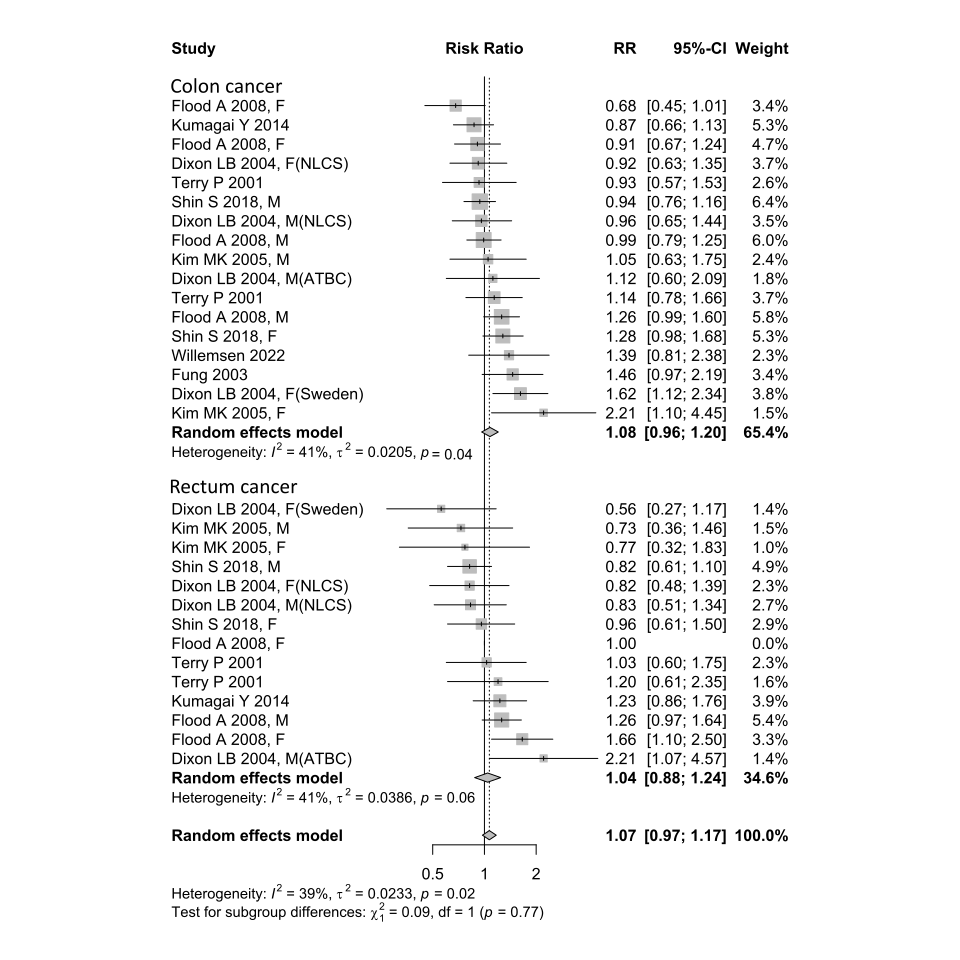


**Figure** S6: Forest plot showed the association between PCA-derived western dietary pattern and colon and rectum cancer


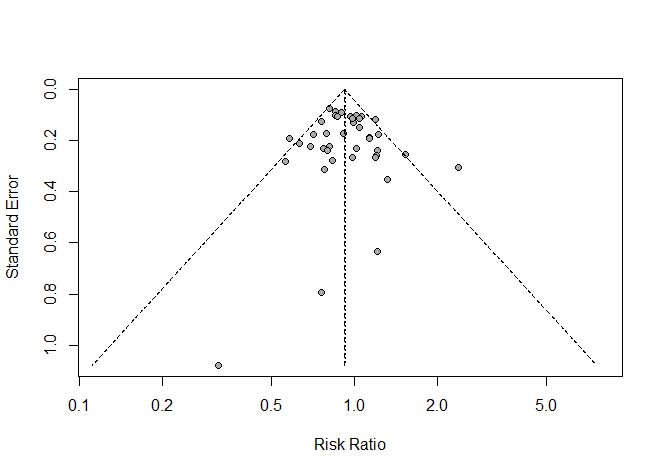


Egger's test: p=0.765

**Figure** S7: Funnel plot to assess the presence of publication bias among the studies included in PCA-derived healthy dietary pattern and GI cancer risk


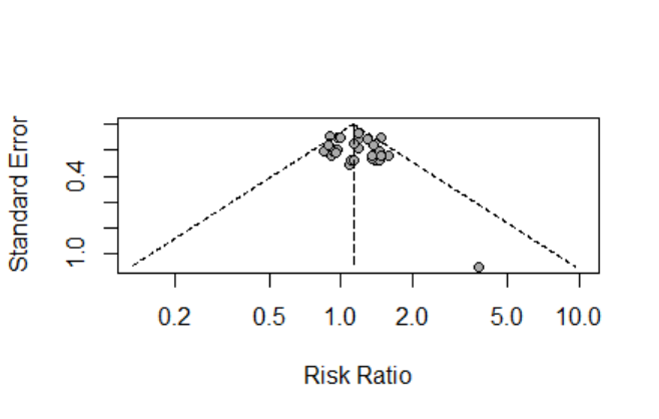


Egger's test: p=0.305

**Figure** S8: Funnel plot to assess Publication bias among those studies included in PCA-derived western dietary patterns and GI cancer risk


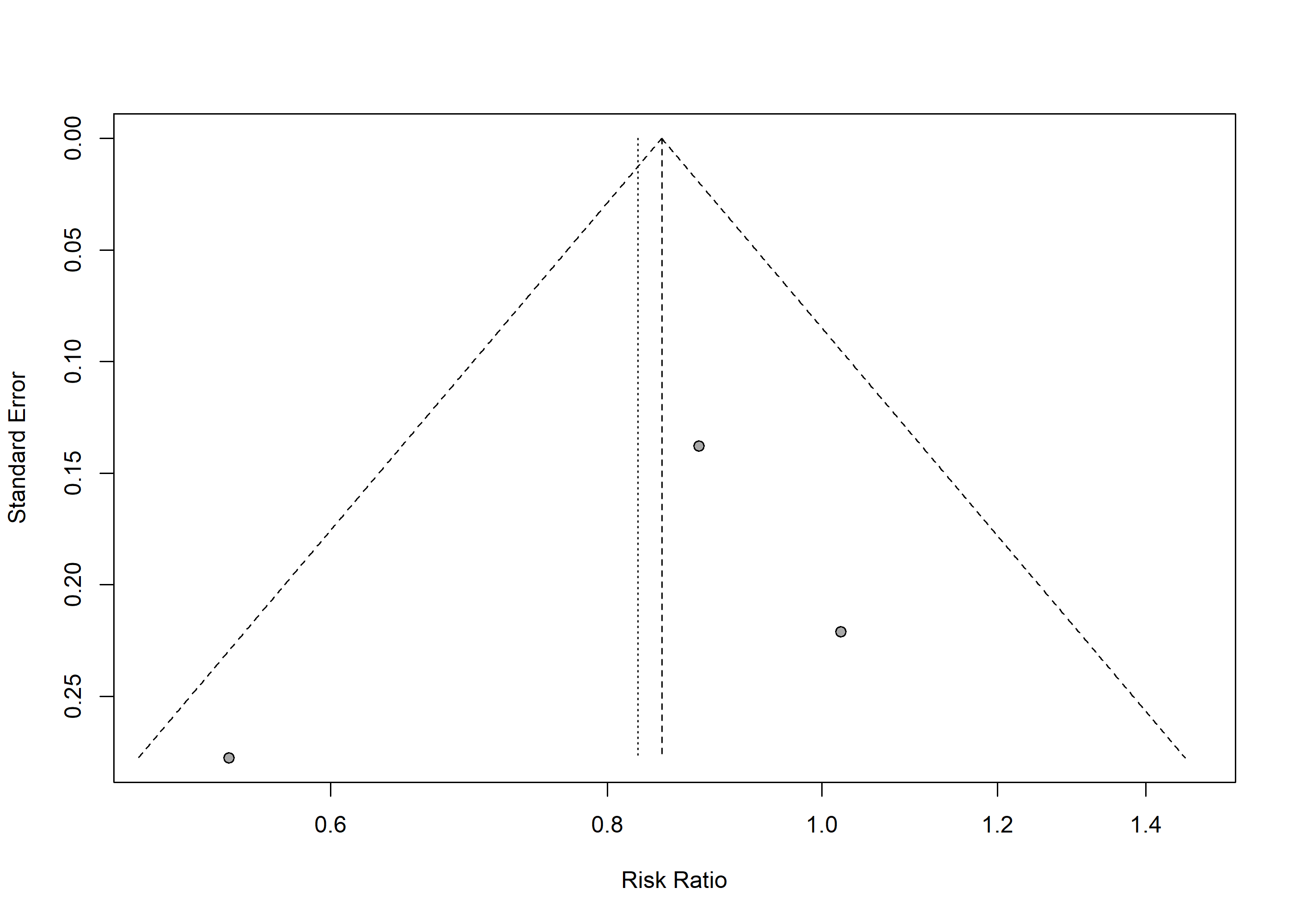


Egger's test, P=0.638)

**Figure** S9: Funnel plot to assess Publication bias among those studies included in RRR-derived healthy dietary patterns and GI cancer risk


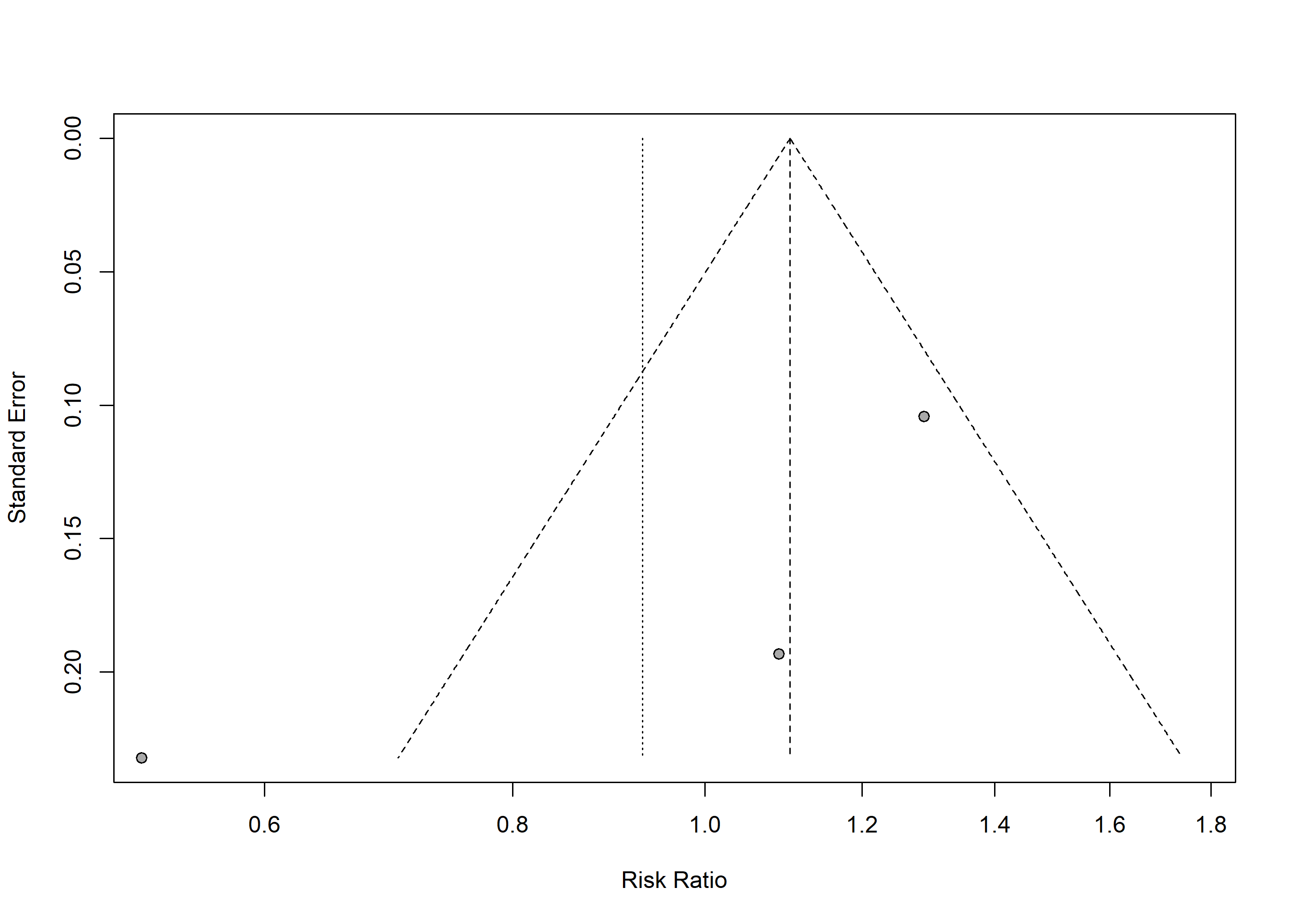


Egger's test, P=0.342

**Figure** S10: Funnel plot to assess Publication bias among those studies included in RRR-derived western dietary patterns and GI cancer risk
